# Supplementary material for: Macrocybin, a Natural Mushroom Triglyceride, Reduces Tumor Growth In Vitro and In Vivo through Caveolin-Mediated Interference with the Actin Cytoskeleton
Source: Molecules. 2020 Dec 18;25(24):6010. doi: 10.3390/molecules25246010 (PMC7766322; doi:10.3390/molecules25246010)
Supplement: Supplementary file 1 [file molecules-25-06010-s001.pdf]

Supplementary Material

Macrocybin, a mushroom natural triglyceride, reduces tumor growth *in vitro* and *in vivo* through caveolin-mediated interference with the actin cytoskeleton

Marcos Vilariño[1], Josune García-Sanmartín[1], Laura Ochoa-Callejero[1], Alberto López-Rodríguez[2], Jaime Blanco-Urgoiti[2], and Alfredo Martínez[1,*]

[1] Oncology Area, Center for Biomedical Research of La Rioja (CIBIR), Logroño, Spain.
[2] CsFlowchem, Campus Universidad San Pablo CEU, Boadilla del Monte, Madrid, Spain.

* Correspondence: amartinezr@riojasalud.es; Tel.: +34 941278775

Index

	Page
1. Natural extract characterization	2
2. Triglyceride synthesis	5
3. Growth modulation activity of synthetic triglycerides	22
4. Comparison of aliphatic carbons between the synthetic triglyceride and the natural extract	25
5. Dataset for xenograft study	26
6. Datasets for qRT-PCR study	29

1. Natural extract characterization

^{1}H-NMR:

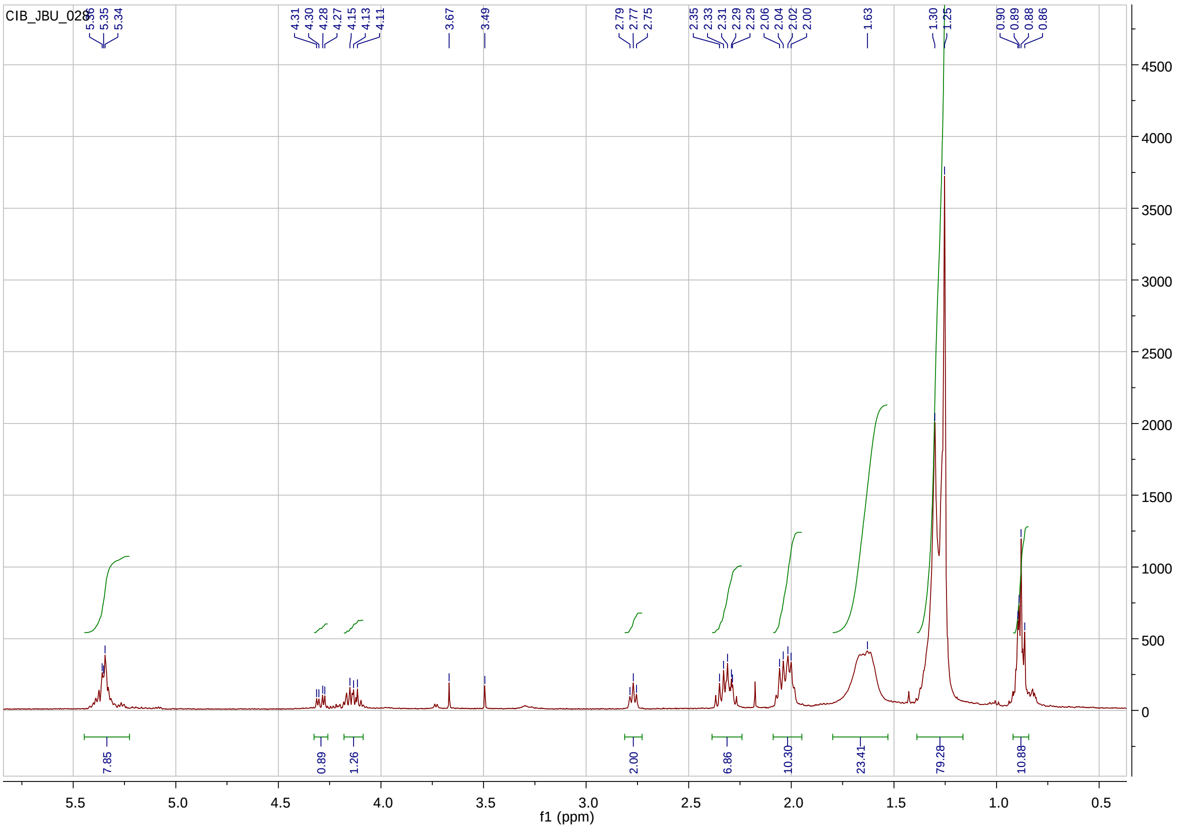

^{13}C-NMR:

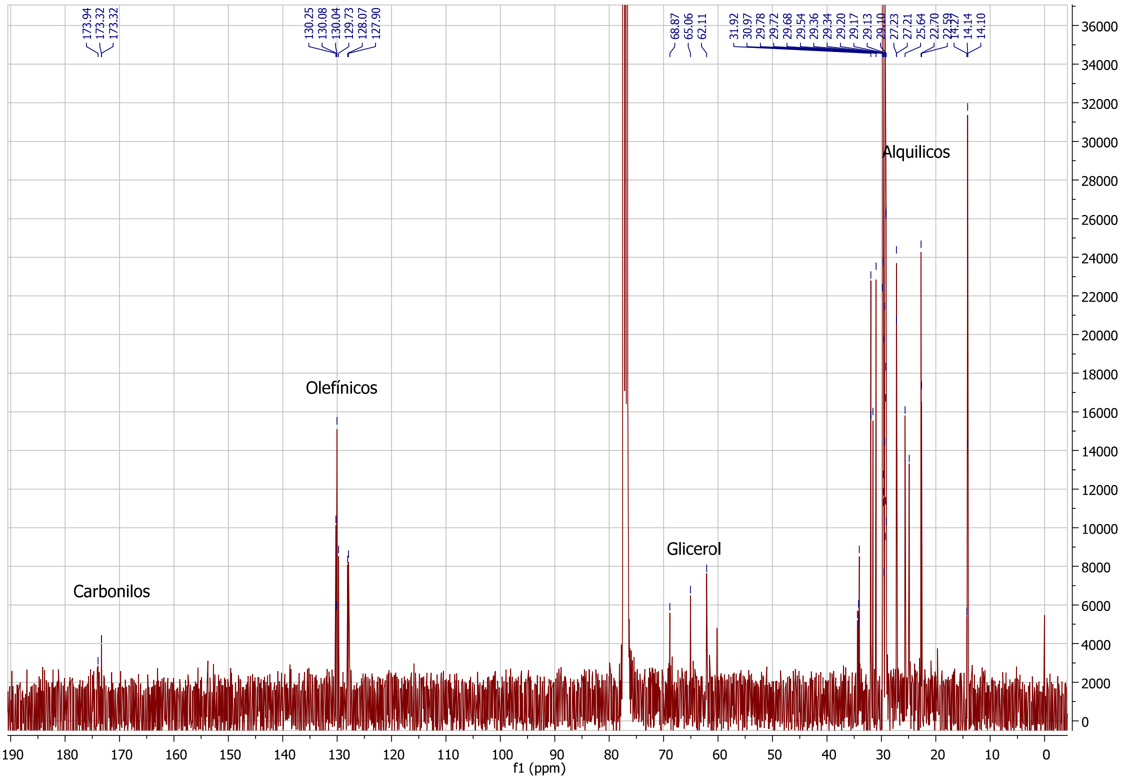

DEPT 135

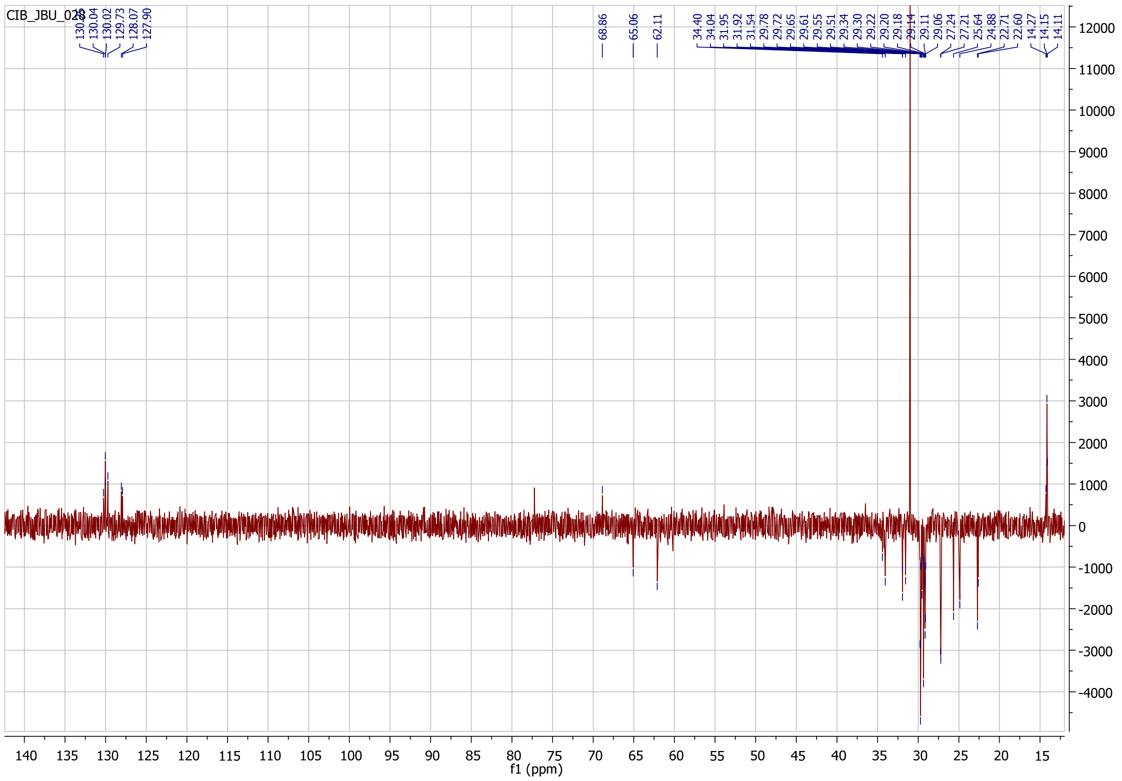

2D-COSY

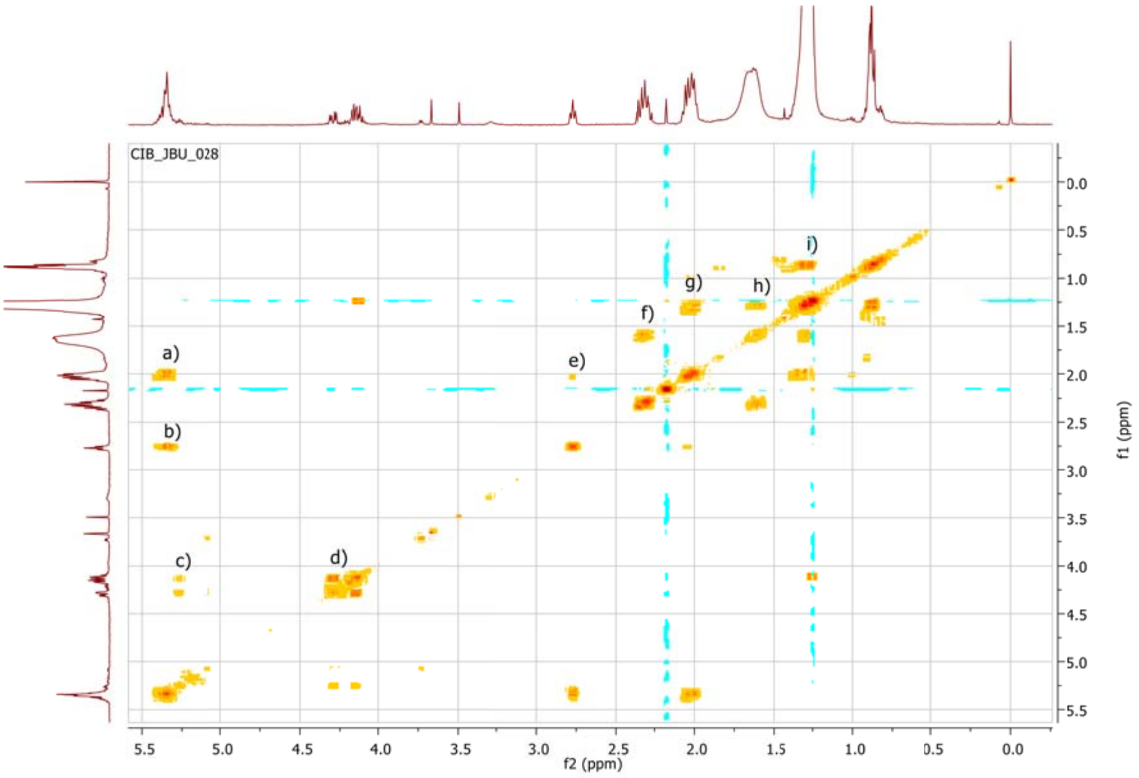
CIB_JBU_028
a)
b)
c)
d)
e)
f)
g)
h)
i)
5.5
5.0
4.5
4.0
3.5
3.0
2.5
2.0
1.5
1.0
0.5
0.0
f2 (ppm)
f1 (ppm)

2. Triglyceride synthesis

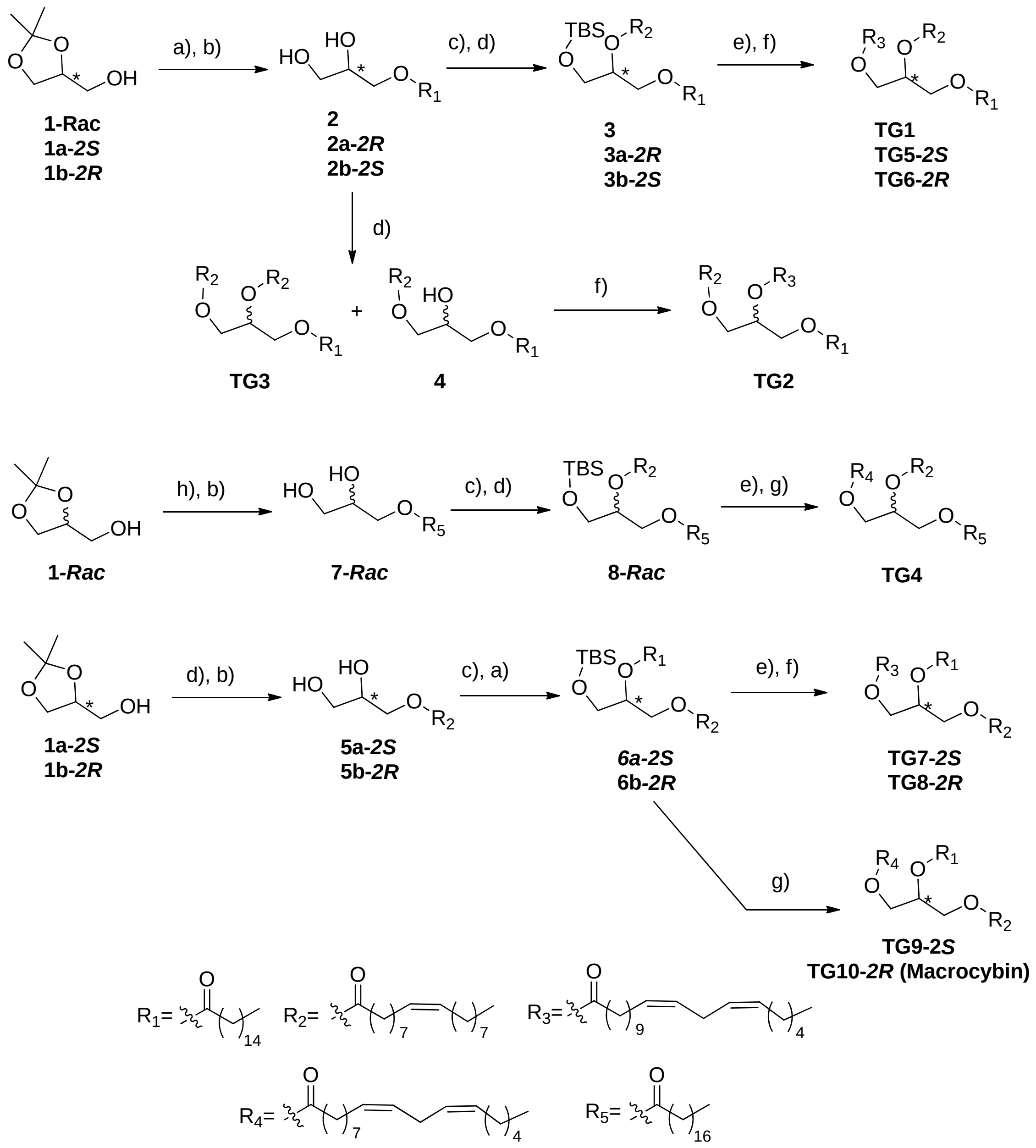

a) Palmitoyl chloride, py, DCM, 0ºC to rt, b)AcOH/H_2O 60ºC, c)TBSCl, Imid, THF, 0ºC to rt, d) oleyl chloride, py, DCM, 0ºC to rt, e) HF·py, py, THF, 0ºC to rt, f) (*11Z,14Z*)-icosa-11,14-dienoic acid, EDCI, DMAP, $CHCl_3$, rt, g) linoleyl chloride, py, DCM, 0ºC to rt, h) stearyl chloride, py, DCM, 0ºC to rt.

Experimental procedure A. Ester formation from acylchloride.

To a solution of the starting alcohol in DCM (8 vol) at 0ºC, pyridine (5 eq) is added followed by the acyl chloride (1.3 eq). The mixture is left at rt overnight. 0.1 N HCl is added (8 vol) and stirred for 5 min at room temperature. The aqueous layer is discarded,

and the organic layer is washed with water and brine, dried over anhydrous MgSO4 filtered and concentrated in vacuo. The crude is used in the following step without further purification.

Experimental procedure B. Acetal hydrolysis.The crude obtained in the step A is suspended in Acetic acid (10 vol) and water (2 vol) and stirred at 60ºC for 3 h. The acetic acid is evaporated under reduced pressure and the aqueous layer is extracted wiDCM. The organic phase is washed with water, 5% sodium bicarbonate in water, more water and brine. The DCM is dried over anhydrous MgSO4 filtered and concentrated in vacuo. The crude of the monoglyceride is used without further purification.

Experimental procedure C. Alcohol protection with Tert-Butyldimethylsilane.

To a solution of the monoglyceride obtained with the procedure B in THF (10 vol) at 0ºC imidazole (1.5 eq) is added followed by Tert-butyldimethylsilyl chloride (1.25eq). The mixture is stirred at rt overnight. The solid is filtered and washed with more THF and the solvent is removed under reduced pressure. The crude is used without further purification.

Experimental procedure D. Alcohol deprotection, silyl cleavage.

To a solution of the diglyceride silylprotected in THF (40 vol) at 0ºCpyridine (3 eq) is added followed by hydrogen fluoride pyridine (3 eq). The reaction is allowed to reach rt. After 3h at rt the mixture is poured into a 5% aqueous solution of sodium bicarbonate. The THF is removed under reduced pressure and the aqueous phase is extracted with MTBE. The organic layer is washed with 1N HCl, water and brine, dried over anhydrous MgSO4 filtered and dried in vacuo. The crude is used without further purification.

Experimental procedure E. Ester formation from the acid and alcohol.

To a solution of the alcohol and the corresponding acid (1.5 eq) in DCM at rt DMAP (30 eq) is added followed by the EDCI (20 eq). The mixture is stirred at rt for 16h. The solid is filtered and washed with more DCM. The solvent is removed under reduced pressure and the crude is purified by flash chromatography using hexane/MTBE mixture as eluent.

TG (C16:0; C18:1, 9z; C20:2, 11z,14z), ***(11Z,14Z)-2-(oleoyloxy)-3-(palmitoyloxy)propyl icosa-11,14-dienoate,*** **TG1.**

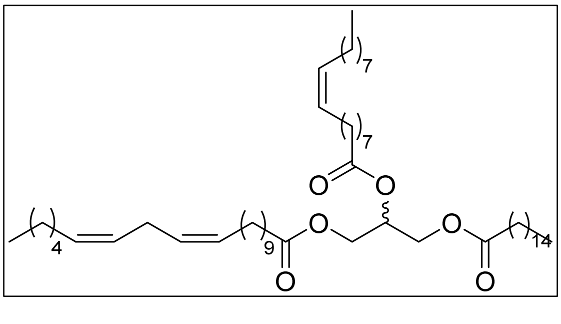

From 200 mg of (±)-(2,2-dimethyl-1,3-dioxolan-4-yl)methanol, following the **procedure A** with palmitoyl chloride, and **procedure B** to obtain **2** with quantitative yield. **2** is transformed following the

procedure C, followed by **procedure A** with oleoyl chloride to obtain 1.08 g of **3** in quantitative yield (2 steps). 95 mg of **TG1** are obtained from 112 mg of **3** following the **procedure D** and **procedure E** with (*11Z,14Z*)-icosa-11,14-dienoic acid after purification by flash chromatography using Hexane/MTBE 3% as eluent (72% yield, 2 steps). ^{1}H NMR (400 MHz, $CDCl_3$) δ 5.42 – 5.28 (m, 6H), 5.26 (td, *J* = 5.9, 3.0 Hz, 1H), 4.29 (dd, *J* = 11.9, 4.3 Hz, 2H), 4.14 (dd, *J* = 11.9, 5.9 Hz, 2H), 2.77 (t, *J* = 6.4 Hz, 2H), 2.31 (td, *J* = 7.6, 2.4 Hz, 6H), 2.10 – 1.94 (m, 8H), 1.67 –1.57 (m, 6H), 1.41 – 1.18 (m, 62H), 0.93 – 0.84 (m, 9H). ^{13}C NMR (101 MHz, $CDCl_3$) δ 173.4, 173.4, 173.0, 130.3, 130.3, 130.2, 129.9, 128.1, 128.1, 69.0, 62.2, 32.1, 32.0, 31.7, 30.3, 29.9, 29.8, 29.8, 29.8, 29.8, 29.7, 29.7, 29.6, 29.6, 29.5, 29.5, 29.4, 29.4, 29.3, 29.2, 27.4, 27.4, 27.3, 27.3, 25.8, 25.0, 25.0, 22.8, 22.7, 14.3, 14.2. Anal. Calcd for C57H104O6 (885.45 g/mol): C, 77.32; H, 11.84%. Found: C, 77.37; H, 11.90%.

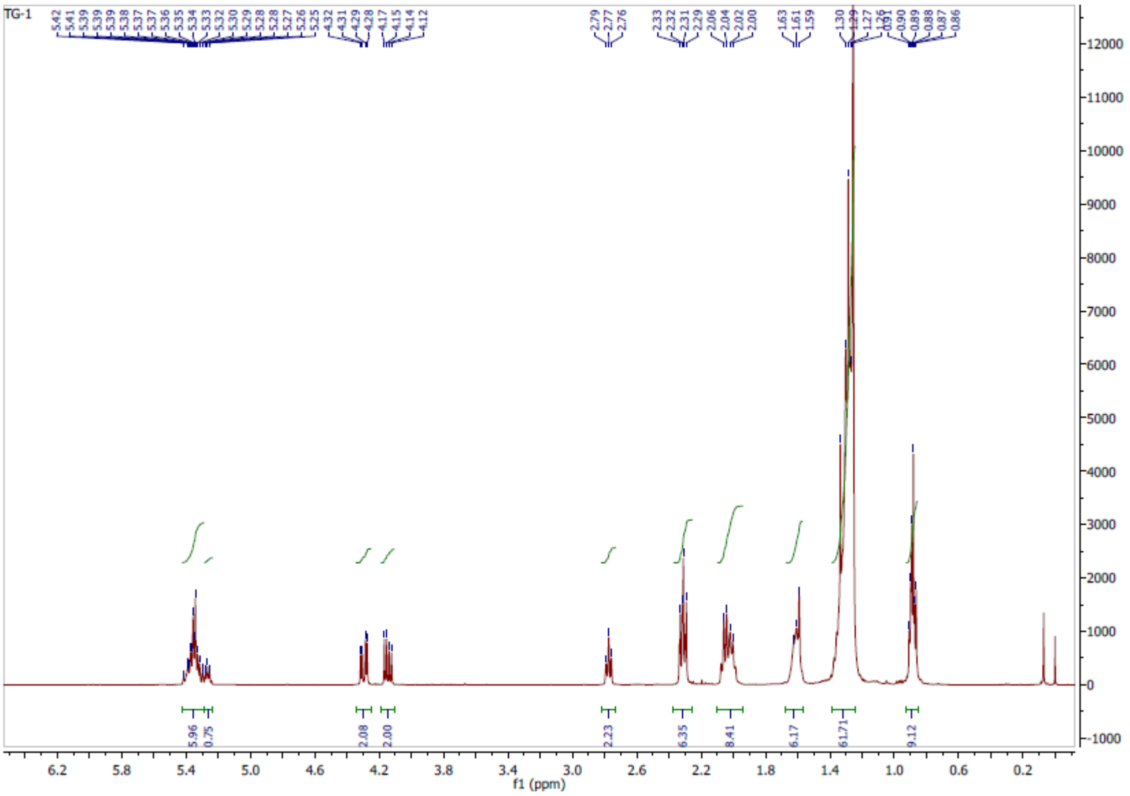

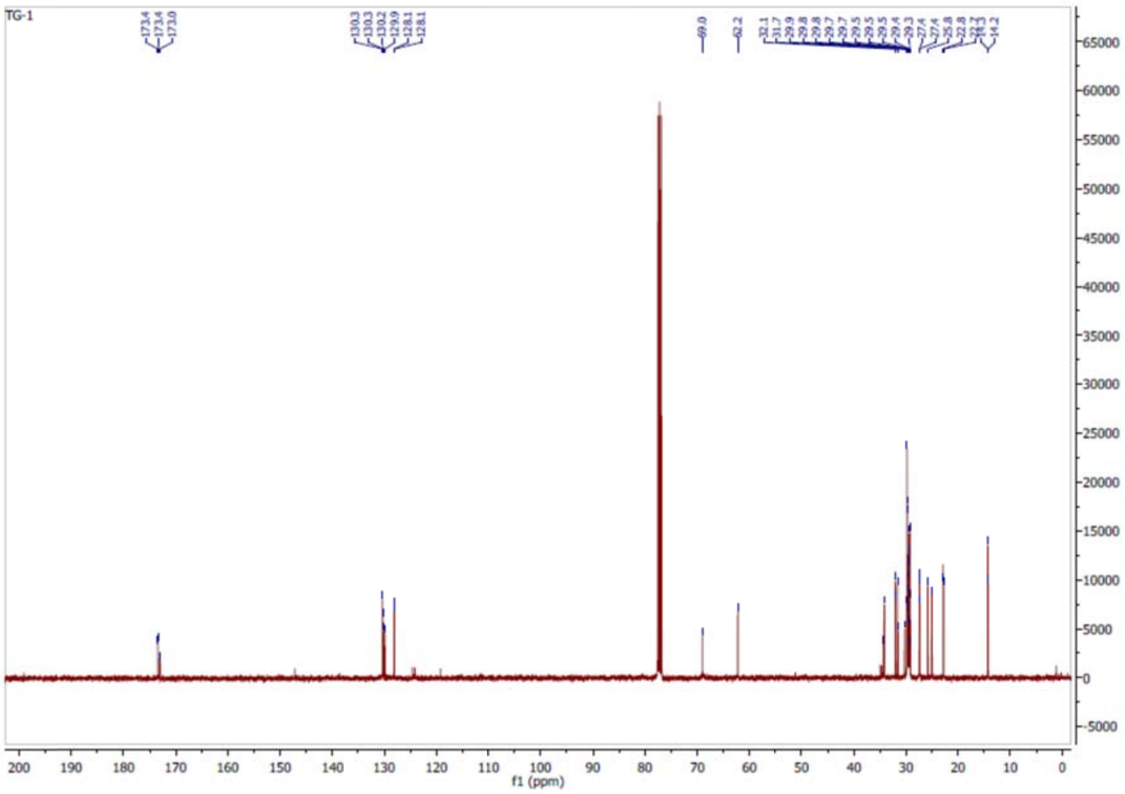

TG (C16:0; C20:2, 11z,14z; C18:1, 9z), ***(11Z,14Z)-1-(oleoyloxy)-3-(palmitoyloxy)propan-2-yl icosa-11,14-dienoate*****, TG2.**

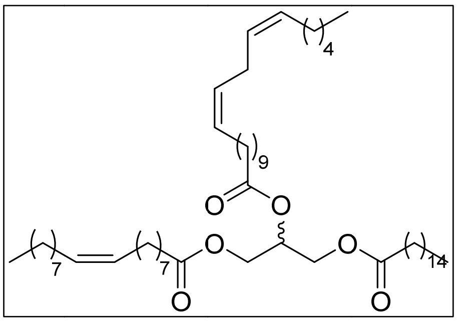

From 86 mg of **4** following the **procedure E** with (11Z,14Z)-icosa-11,14-dienoic acid 95 mg of **TG2** are obtained after purification by flash chromatography using Hexane/MTBE 3% as eluent (74% yield). ^{1}H NMR (400 MHz, CDCl3) δ 5.40 – 5.30 (m, 6H), 5.26 (tt, *J* = 5.9, 4.3 Hz, 1H), 4.29 (dd, *J* = 11.9, 4.3 Hz, 2H), 4.14 (dd, *J* = 11.9, 6.0 Hz, 2H), 2.77 (t, *J* = 6.4 Hz, 2H), 2.31 (td, *J* = 7.6, 2.5 Hz, 6H), 2.10-1.96 (m, 8H), 1.66 – 1.57 (m, 6H), 1.39 – 1.19 (m, 62H), 0.88 (t, *J* = 6.8 Hz, 9H). ^{13}C NMR (101 MHz, $CDCl_3$) δ 173.4, 173.4, 173.0, 130.3, 130.2, 130.1, 129.8, 128.1, 128.1, 69.0, 62.2, 34.3, 34.1, 34.1, 32.1, 32.0, 31.8, 29.9, 29.8, 29.8, 29.8, 29.8, 29.7, 29.6, 29.5, 29.5, 29.5, 29.4, 29.4, 29.4, 29.3, 29.2, 29.2, 27.4, 27.3, 25.8, 25.0, 25.0, 24.9, 22.8, 22.7, 14.2, 14.2. Anal. Calcd for C57H104O6 (885.45 g/mol): C, 77.32; H, 11.84%. Found: C, 77.38; H, 11.79%.

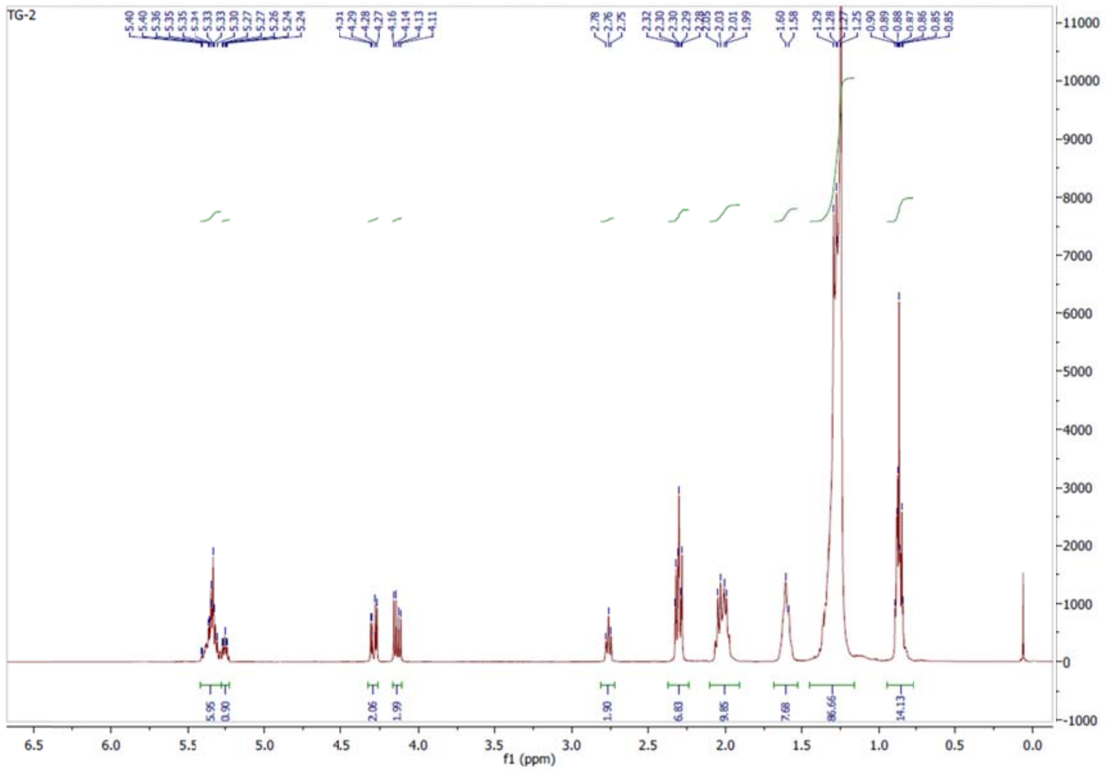
TG-2
f1 (ppm)

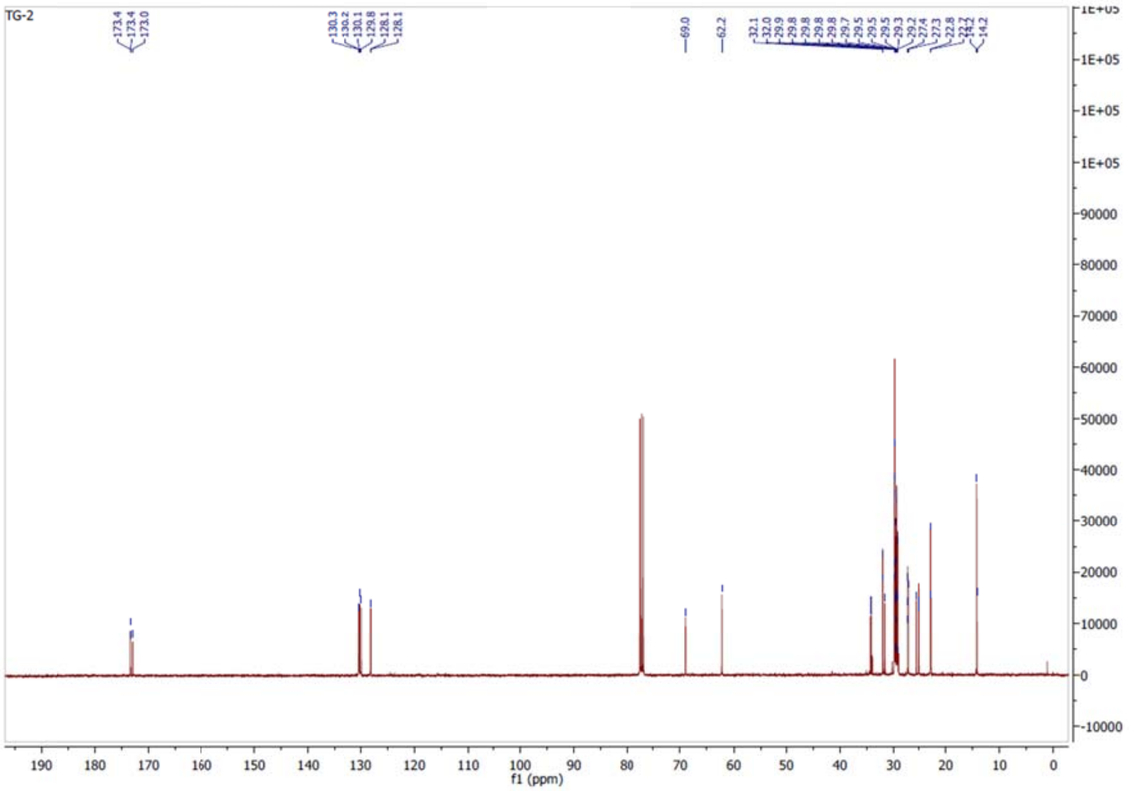
TG-2
f1 (ppm)

TG (C16:0; C18:1, 9z; C18:1, 9z), (*Z*)*-3-(palmitoyloxy)propane-1,2-diyl dioleate*, TG3.

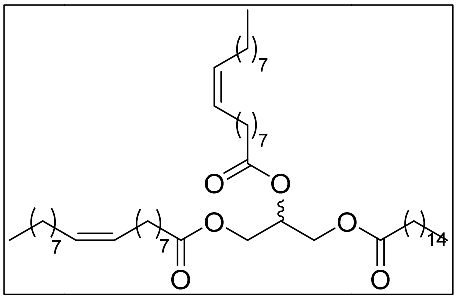

From 500 mg of **2** following the **procedure A** with oleoyl chloride 150 mg of **TG3** are obtained after purification by flash chromatography using Hexane/MTBE 3% as eluent (8% yield). In this step 540 mg of **4** is obtained as a colorless oil (60% yield) ^{1}H NMR (400 MHz, $CDCl_3$) δ 5.39 – 5.30 (m, 4H), 5.26 (tt, *J* = 5.9, 4.3 Hz, 1H), 4.29 (dd, *J* = 11.9, 4.3 Hz, 2H), 4.14 (dd, *J* = 11.9, 6.0 Hz, 2H), 2.31 (td, *J* = 7.6, 2.5 Hz, 6H), 2.01 (q, *J* = 5.9, 5.3 Hz, 8H), 1.66 – 1.57 (m, 6H), 1.39 – 1.19 (m, 64H), 0.86-0.90 (m, 9H). ^{13}C NMR (101 MHz, $CDCl_3$) δ 173.4, 173.4, 173.0, 130.3, 130.2, 130.1, 129.8, 69.0, 62.2, 34.3, 34.1, 34.1, 32.1, 32.0, 31.8, 29.9, 29.8, 29.8, 29.8, 29.8, 29.7, 29.6, 29.6, 29.5, 29.5, 29.4, 29.4, 29.4, 29.3, 29.2, 29.2, 27.4, 27.3, 25.8, 25.0, 25.0, 24.9, 22.8, 22.7, 14.3, 14.2. Anal. Calcd for C55H102O6 (859.42 g/mol): C, 76.87; H, 11.96%. Found: C, 76.94; H, 12.01%.

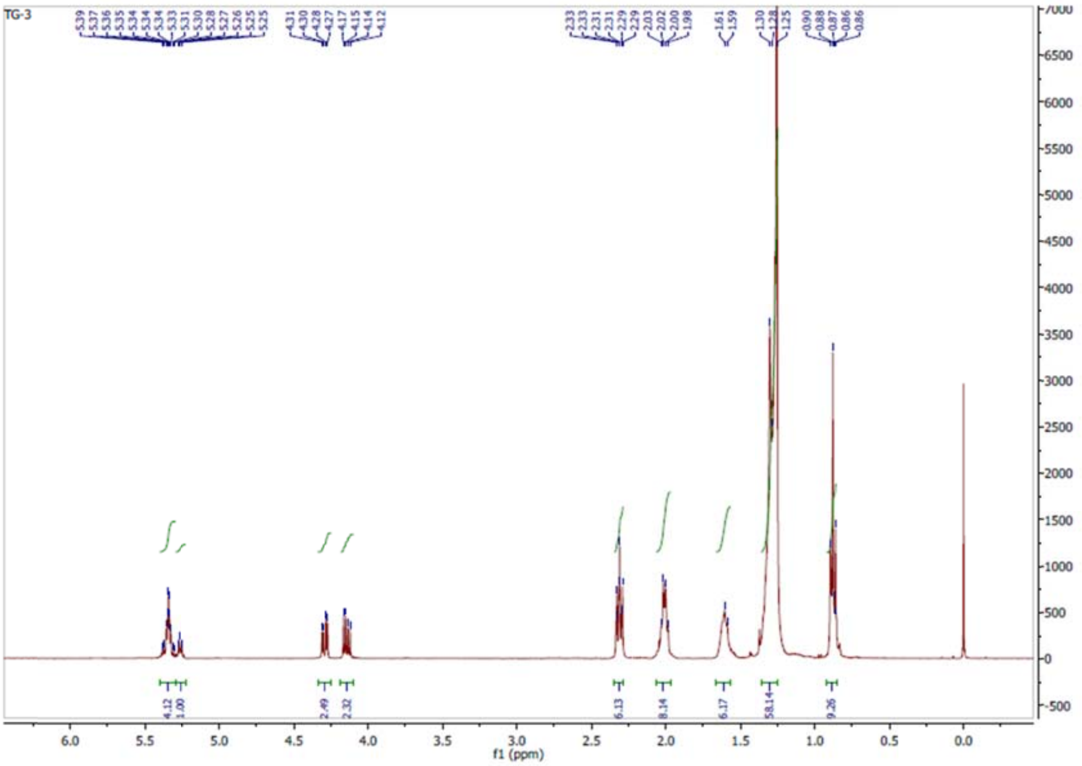

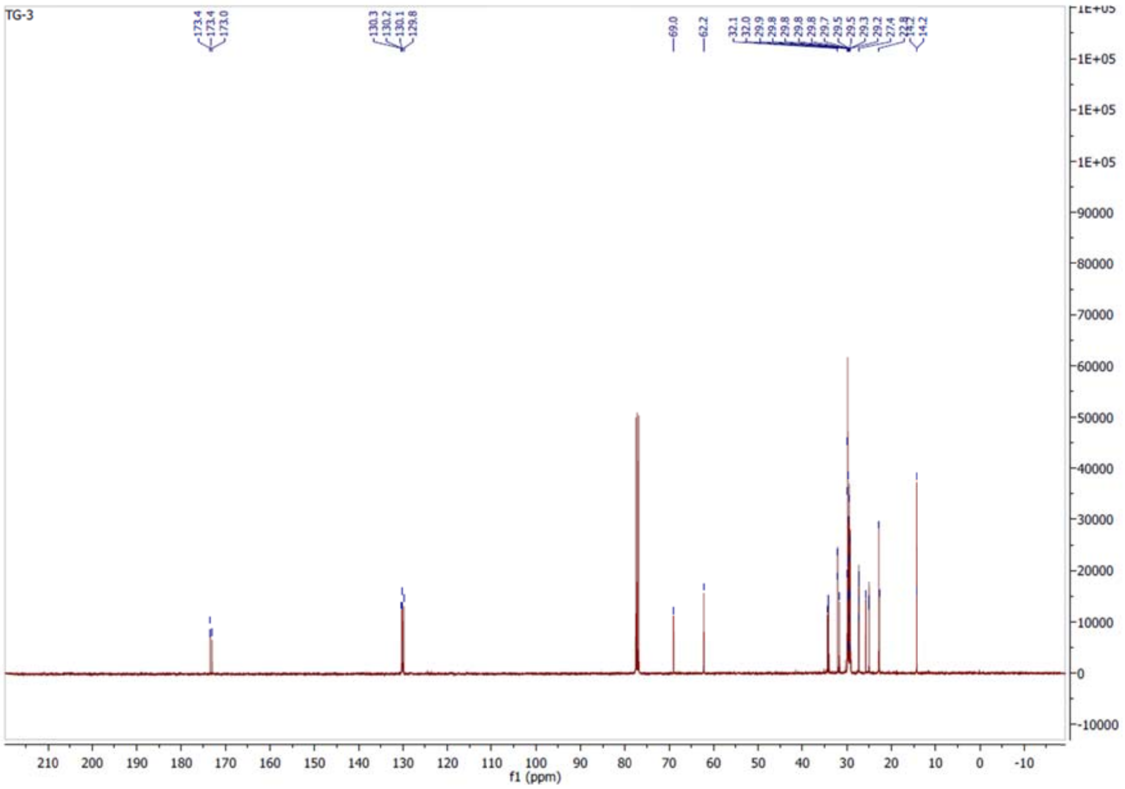

TG (C18:0; C18:1, 9z; C18:2, 9z,12z), ***(9Z,12Z)-2-(oleoyloxy)-3-(stearoyloxy)propyl octadeca-9,12-dienoate*, TG4.**

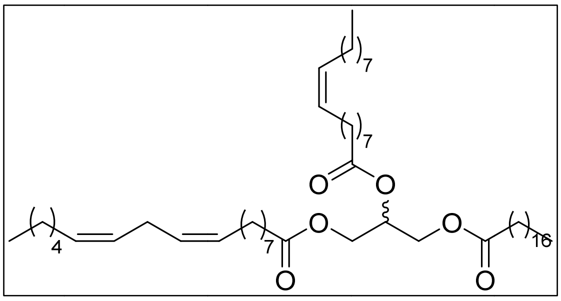

From 1 g of(±)-(2,2-dimethyl-1,3-dioxolan-4-yl)methanol, following the **procedure A** with stearic chloride, and **procedure B** to obtain **7**, which is transformed following the **procedure C**, followed by **procedure A** with oleoyl chloride to obtain 2.95 g of **8** (53%, 2 steps). 67 mg of **TG4** are obtained from 110 mg of **8** following the **procedure D** and **procedure A** with (*9Z*,*12Z*)-octadeca-9,12-dienoyl chloride after purification by flash chromatography using Hexane/MTBE 2% as eluent (62% yield, 2 steps). ^{1}H NMR (400 MHz, $CDCl_3$) δ 5.41 – 5.29 (m, 6H), 5.26 (tt, *J* = 5.9, 4.6 Hz, 1H), 4.29 (dd, *J* = 11.9, 4.3 Hz, 2H), 4.14 (dd, *J* = 11.9, 6.0 Hz, 2H), 2.77 (t, *J* = 6.5 Hz, 2H), 2.31 (td, *J* = 7.5, 2.4 Hz, 6H), 2.07 – 1.98 (m, 8H), 1.65 – 1.57 (m, 4H), 1.38 – 1.22 (m, 64H), 0.90 – 0.86 (m, 9H). ^{13}C NMR (101 MHz, $CDCl_3$) δ 173.4 173.4, 173.0, 130.3, 130.2, 130.1, 129.8, 128.2, 128.0, 69.0, 62.2, 34.3, 34.2, 34.1, 32.1, 32.0, 31.7, 29.9, 29.8, 29.8, 29.7, 29.7, 29.5, 29.5, 29.5, 29.4, 29.4, 29.4, 29.3, 29.3, 29.2, 29.2, 29.1, 27.3, 27.3, 25.8, 24.9, 24.9, 22.8, 22.7, 14.3, 14.2. Anal. Calcd for C57H104O6 (885.45 g/mol): C, 77.32; H, 11.84%. Found: C, 77.26; H, 11.79%.

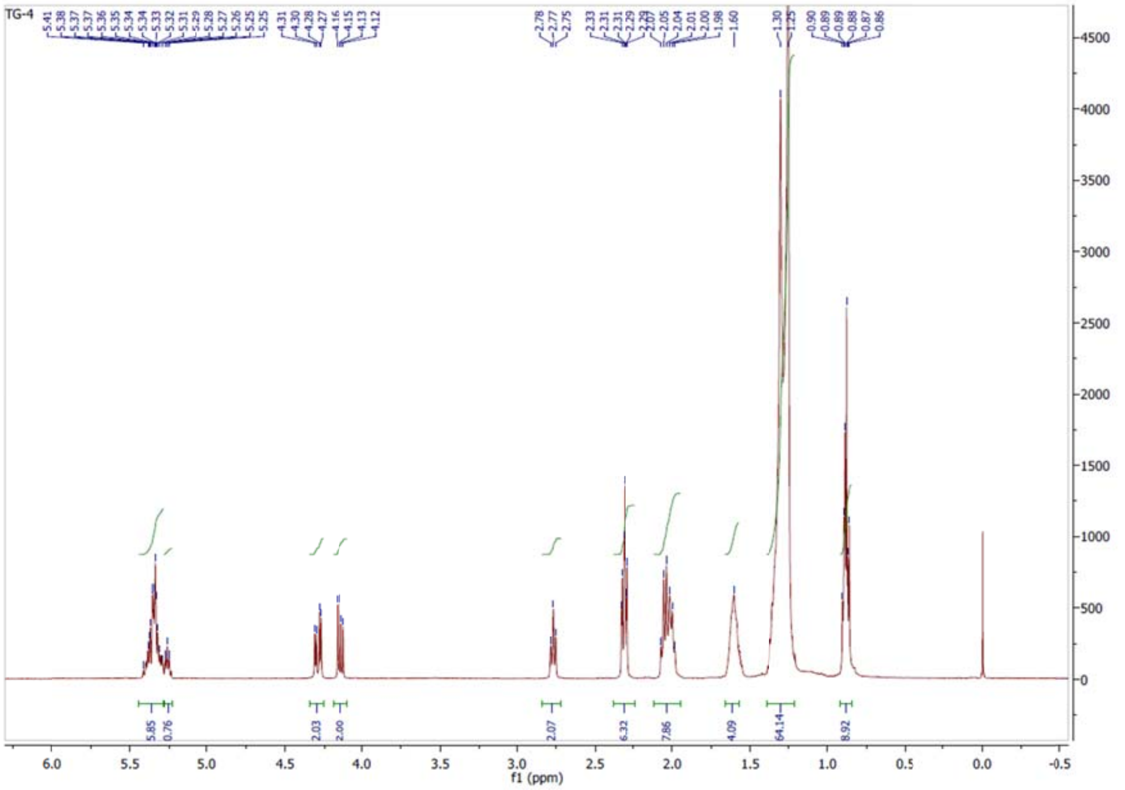
TG-4
f1 (ppm)
5.85
0.76
2.03
2.00
2.07
6.32
7.86
4.09
64.14
8.92

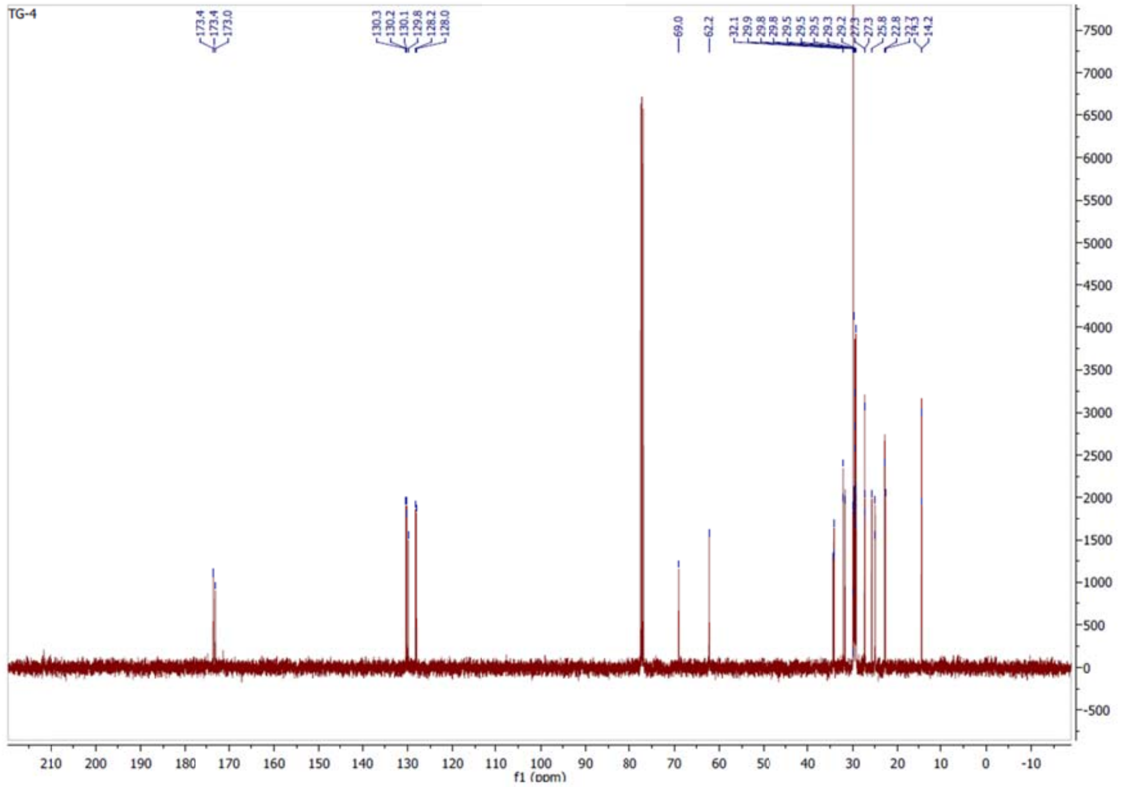
TG-4
173.4
173.4
173.0
130.3
130.2
130.1
129.8
128.2
128.0
69.0
62.2
f1 (ppm)

***2S*-TG (C16:0; C18:1, 9z; C20:2, 11z,14z), *(11Z,14Z)-(S)-2-(oleoyloxy)-3-(palmitoyloxy)propyl icosa-11,14-dienoate*, TG5.**

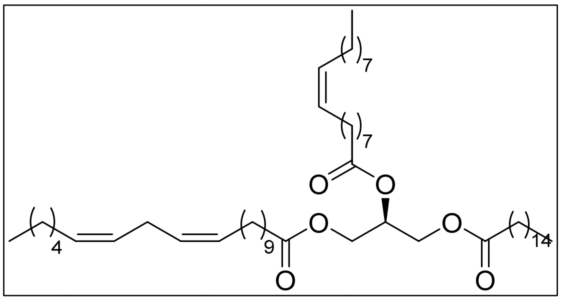

From 500 mg of (S)-(2,2-dimethyl-1,3-dioxolan-4-yl)methanol, following the **procedure A** with palmitoyl chloride, and **procedure B** to obtain **2a** with quantitative yield. **2a** is transformed following the **procedure C**, followed by **procedure A** with oleoyl chloride to obtain, after purification by flash chromatography using Hexane/MTBE 3% as eluent, 1.56 g of **3a** (58% yield, 4 steps). 88 mg of **TG5** is obtained from 76 mg of **3a** following the **procedure D** and **procedure E** with (11Z,14Z)-icosa-11,14-dienoic acid after purification by flash chromatography using Hexane/MTBE 3% as eluent (92% yield, 2 steps). ^{1}H NMR (400 MHz, $CDCl_3$) δ 5.41 – 5.30 (m, 6H), 5.26 (td, *J* = 5.9, 3.0 Hz, 1H), 4.29 (dd, *J* = 11.9, 4.3 Hz, 2H), 4.14 (dd, *J* = 11.9, 5.9 Hz, 2H), 2.77 (t, *J* = 6.4 Hz, 2H), 2.31 (td, *J* = 7.6, 2.4 Hz, 6H), 2.10 – 1.94 (m, 8H), 1.67 – 1.57 (m, 6H), 1.41 – 1.18 (m, 62H), 0.93 – 0.84 (m, 9H). ^{13}C NMR (101 MHz, $CDCl_3$) δ 173.4, 173.4, 173.0, 130.3, 130.2, 130.1, 129.8, 128.1, 128.0, 69.0, 62.2, 34.2, 32.0, 32.1, 31.7, 30.3, 29.9, 29.8, 29.8, 29.8, 29.7, 29.6, 29.6, 29.5, 29.5, 29.5, 29.4, 29.4, 29.4, 29.3, 29.2, 27.3, 27.3, 27.4, 27.3, 25.8, 25.0, 25.0, 22.8, 22.7, 14.3, 14.2. Anal. Calcd for C57H104O6 (885.45 g/mol): C, 77.32; H, 11.84%. Found: C, 77.27; H, 11.90%.

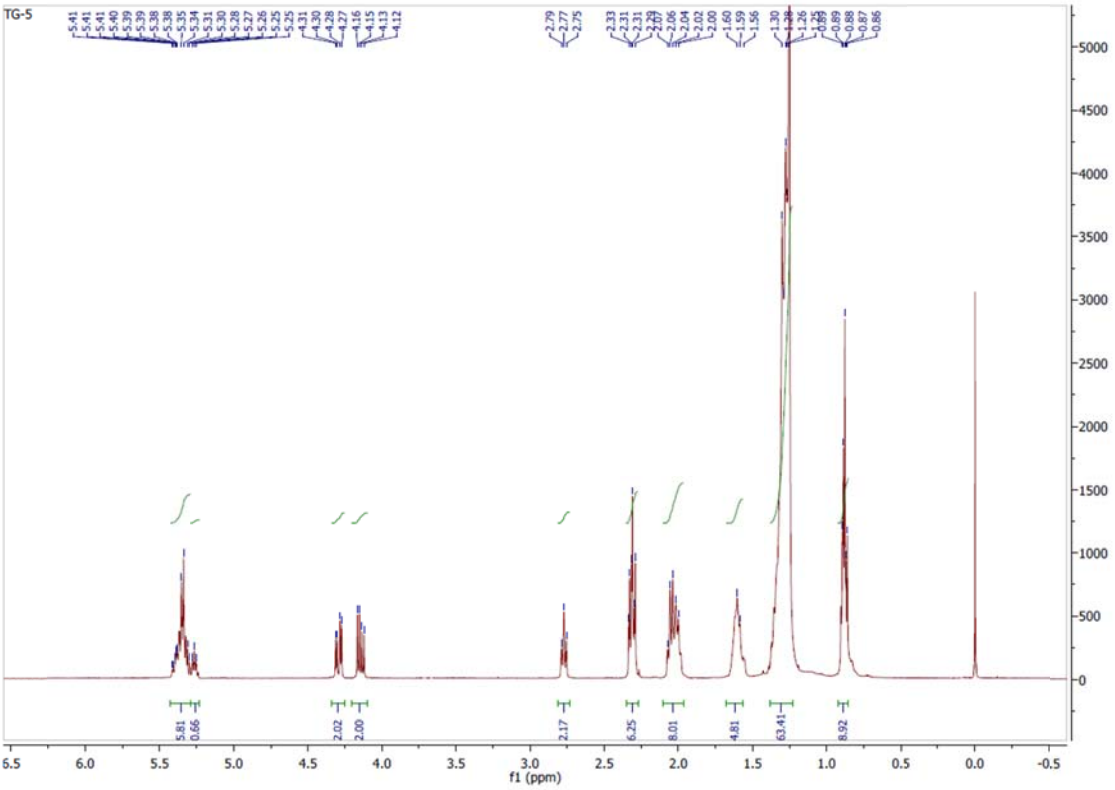

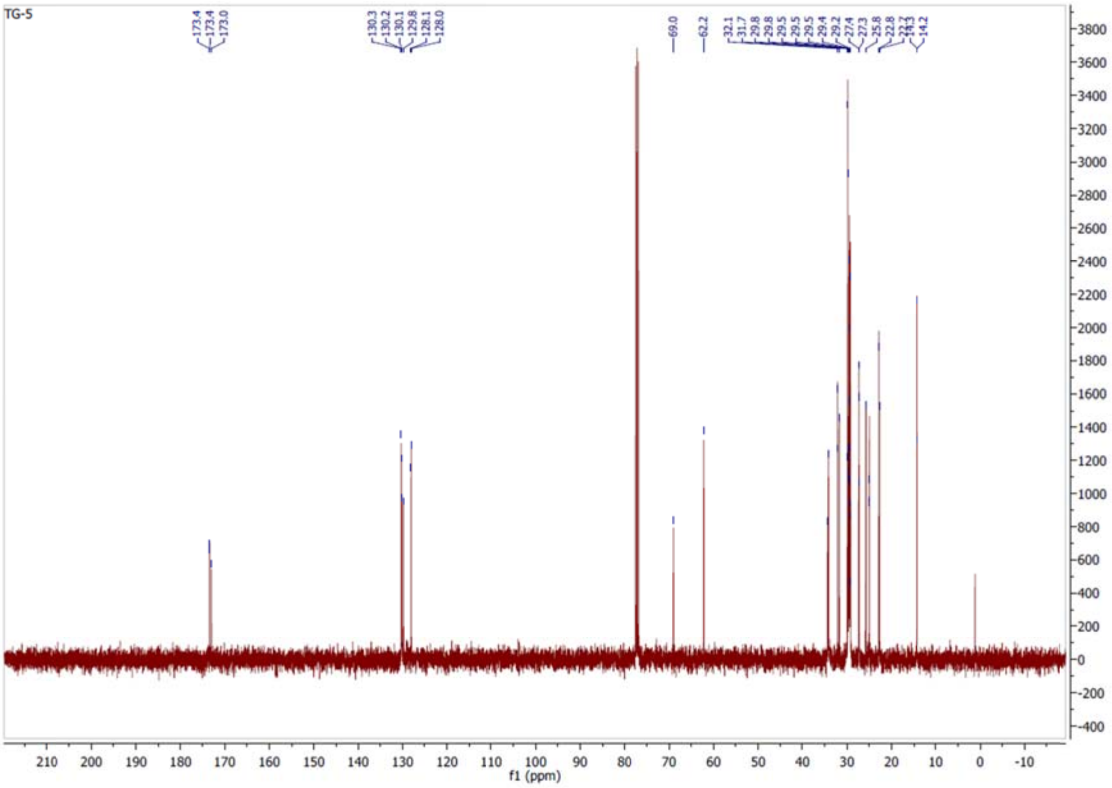

***2R*-TG (C16:0; C18:1, 9z; C20:2, 11z,14z), *(11Z,14Z)-(S)-2-(oleoyloxy)-3-(palmitoyloxy)propyl icosa-11,14-dienoate*, TG6.**

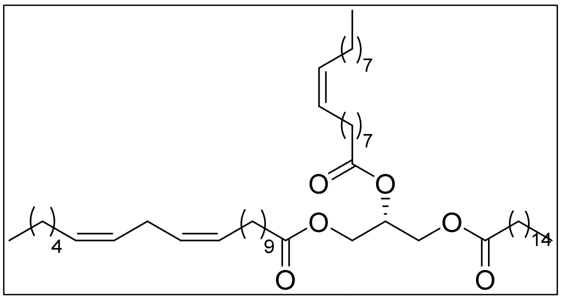

From 431 mg of (*R*)-(2,2-dimethyl-1,3-dioxolan-4-yl)methanol, following the **procedure A** with palmitoyl chloride, and **procedure B** to obtain **2b** with quantitative yield. **2b** is transformed following the **procedure C**, followed by **procedure A** with oleoyl chloride to obtain, after purification by flash chromatography using Hexane/MTBE 3% as eluent, 1.27 g of **3b** (55% yield, 4 steps). 77 mg of **TG6** is obtained from 76 mg of **3b** following the **procedure D** and **procedure E** with (11Z,14Z)-icosa-11,14-dienoic acid after purification by flash chromatography using Hexane/MTBE 3% as eluent (81% yield, 2 steps). ^{1}H NMR (400 MHz, $CDCl_3$) δ 5.42 – 5.28 (m, 6H), 5.26 (td, *J* = 5.9, 3.0 Hz, 1H), 4.29 (dd, *J* = 11.9, 4.3 Hz, 2H), 4.14 (dd, *J* = 11.9, 5.9 Hz, 2H), 2.77 (t, *J* = 6.4 Hz, 2H), 2.31 (td, *J* = 7.6, 2.4 Hz, 6H), 2.10 – 1.94 (m, 8H), 1.67 – 1.57 (m, 6H), 1.41 – 1.18 (m, 62H), 0.93 – 0.84 (m, 9H). ^{13}C NMR (101 MHz, $CDCl_3$) δ 173.4, 173.4, 173.0, 130.3, 130.2, 130.1, 129.8, 128.1, 128.0, 69.0, 62.2, 34.2, 32.1, 32.0, 31.7, 30.3, 29.9, 29.8, 29.8, 29.8, 29.7, 29.6, 29.6, 29.6, 29.5, 29.5, 29.4, 29.4, 29.4, 29.3, 29.2, 27.3, 27.3, 27.4, 27.3, 25.8, 25.0, 25.0, 22.8, 22.7, 14.3, 14.2. Anal. Calcd for C57H104O6 (885.45 g/mol): C, 77.32; H, 11.84%. Found: C, 77.36; H, 11.89%.

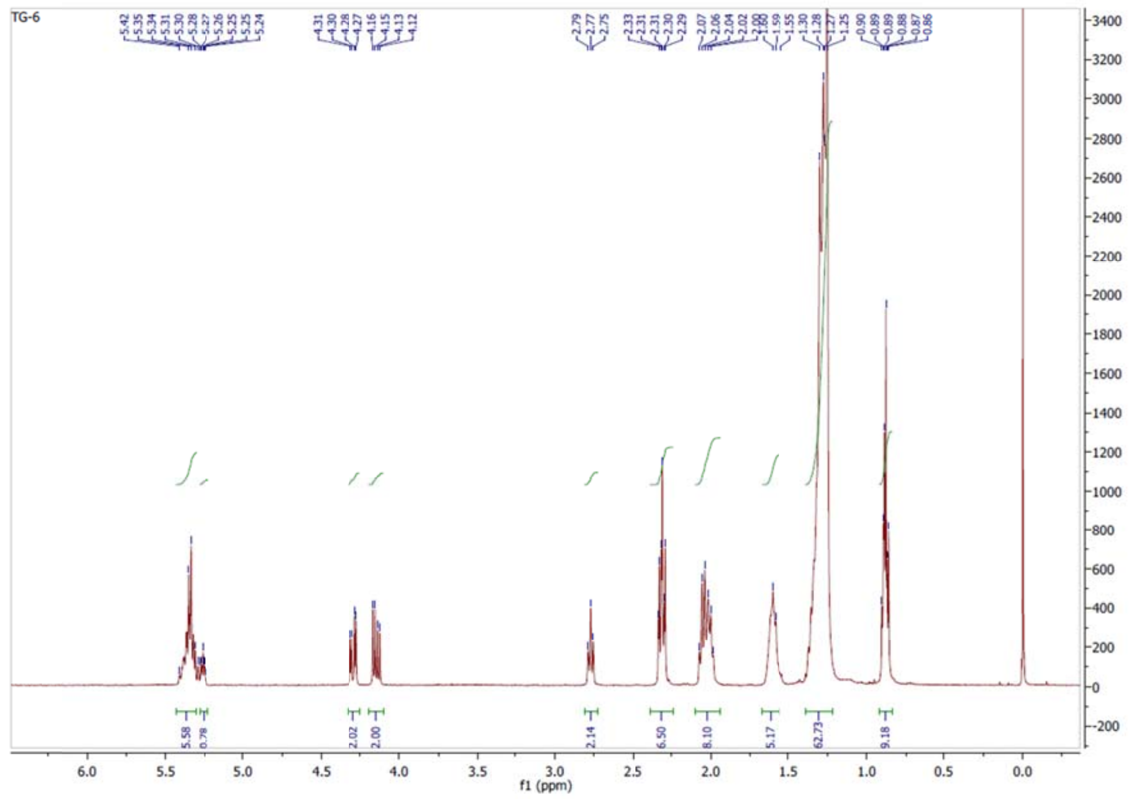
TG-6
f1 (ppm)

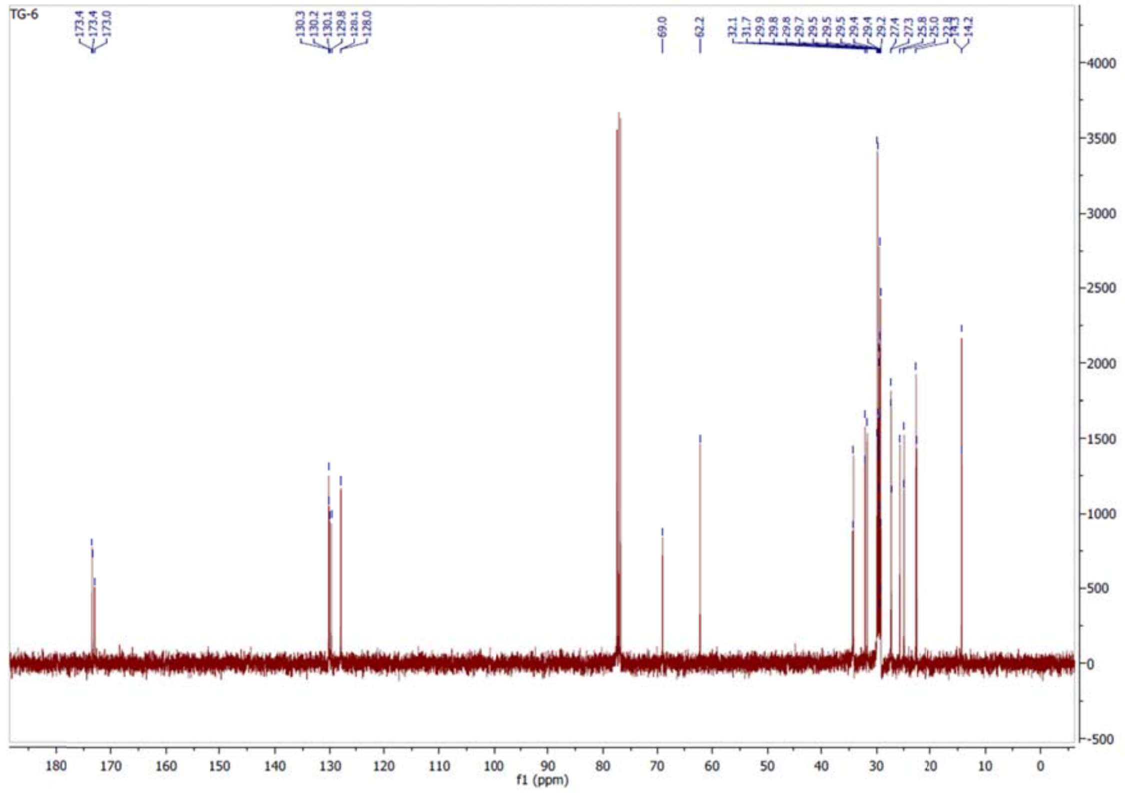
TG-6
f1 (ppm)

***2S*-TG (C20:2, 11z,14z; C16:0; C18:1, 9z), *(11Z,14Z)-(S)-3-(oleoyloxy)-2-(palmitoyloxy)propyl icosa-11,14-dienoate*, TG7.**

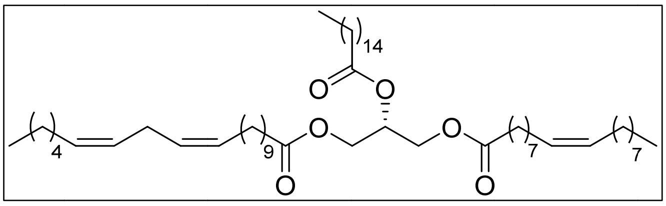

From 500 mg of (*R*)-(2,2-dimethyl-1,3-dioxolan-4-yl)methanol, following the **procedure A** with oleyl chloride, and **procedure B** to obtain **5a** with quantitative yield. **5a** is transformed following the **procedure C**, followed by **procedure A** with palmitoyl chloride to obtain, after purification by flash chromatography using Hexane/MTBE 1% as eluent, 1.43 g of **6a** (53% yield, 4 steps). 90 mg of **TG7** are obtained from 77 mg of **6a** following the **procedure D** and **procedure E** with (11Z,14Z)-icosa-11,14-dienoic acid after purification by flash chromatography using Hexane/MTBE 3% as eluent (95% yield, 2 steps). ^{1}H NMR (400 MHz, $CDCl_3$) δ 5.41 – 5.29 (m, 6H), 5.27 (tt, *J* = 6.0, 4.4 Hz, 1H), 4.29 (dd, *J* = 11.9, 4.3 Hz, 2H), 4.14 (dd, *J* = 11.9, 6.0 Hz, 2H), 2.77 (t, *J* = 6.5 Hz, 2H), 2.31 (td, *J* = 7.5, 2.4 Hz, 6H), 2.10 – 1.96 (m, 8H), 1.66 – 1.56 (m, 6H), 1.37 – 1.21 (m, 62H), 0.93 – 0.84 (m, 9H). ^{13}C NMR (101 MHz, $CDCl_3$) δ 173.3, 173.3, 172.9, 130.3, 130.2, 130.1, 129.8, 128.1, 128.0, 69.0, 62.2, 34.3, 34.1, 32.1, 32.0, 31.7, 29.9, 29.8, 29.8, 29.7, 29.6, 29.5, 29.5, 29.4, 29.4, 29.4, 29.3, 29.2, 29.2, 27.3, 27.3, 27.2, 25.7, 25.0, 24.9, 22.8, 22.7, 14.2, 14.2. Anal. Calcd for C57H104O6 (885,43 g/mol): C, 77.32; H, 11.84% Found: C, 77.13; H, 11.75%.

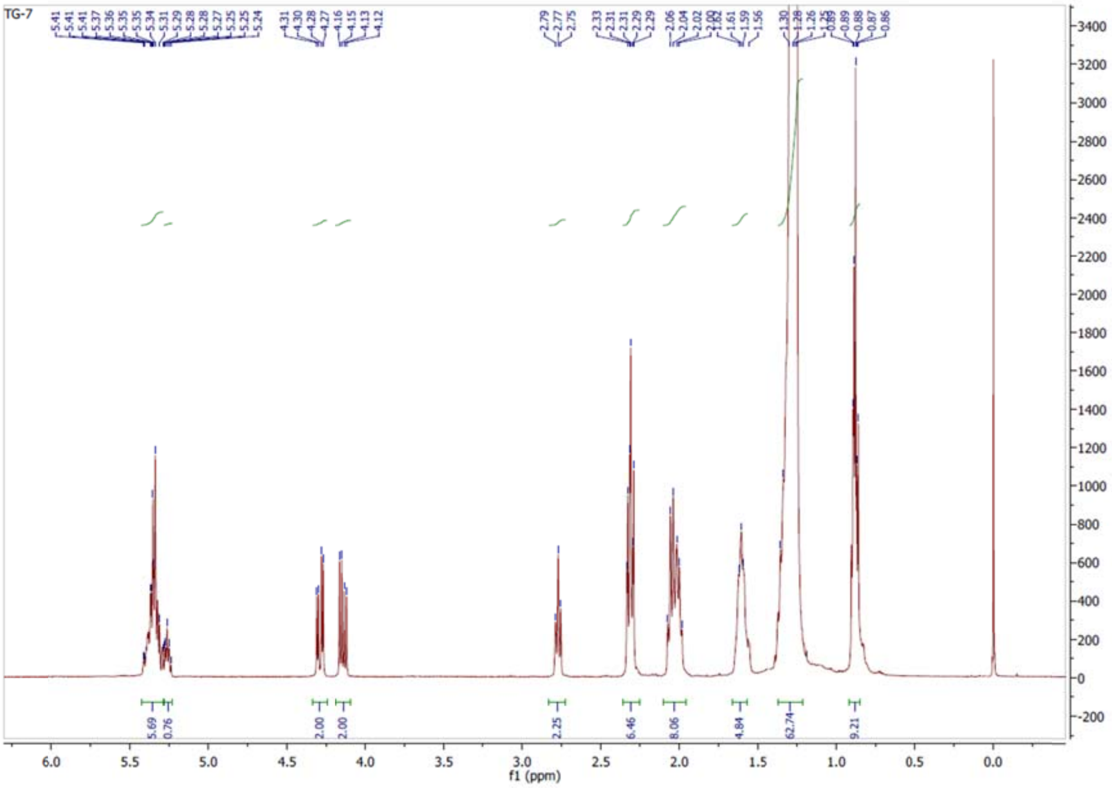

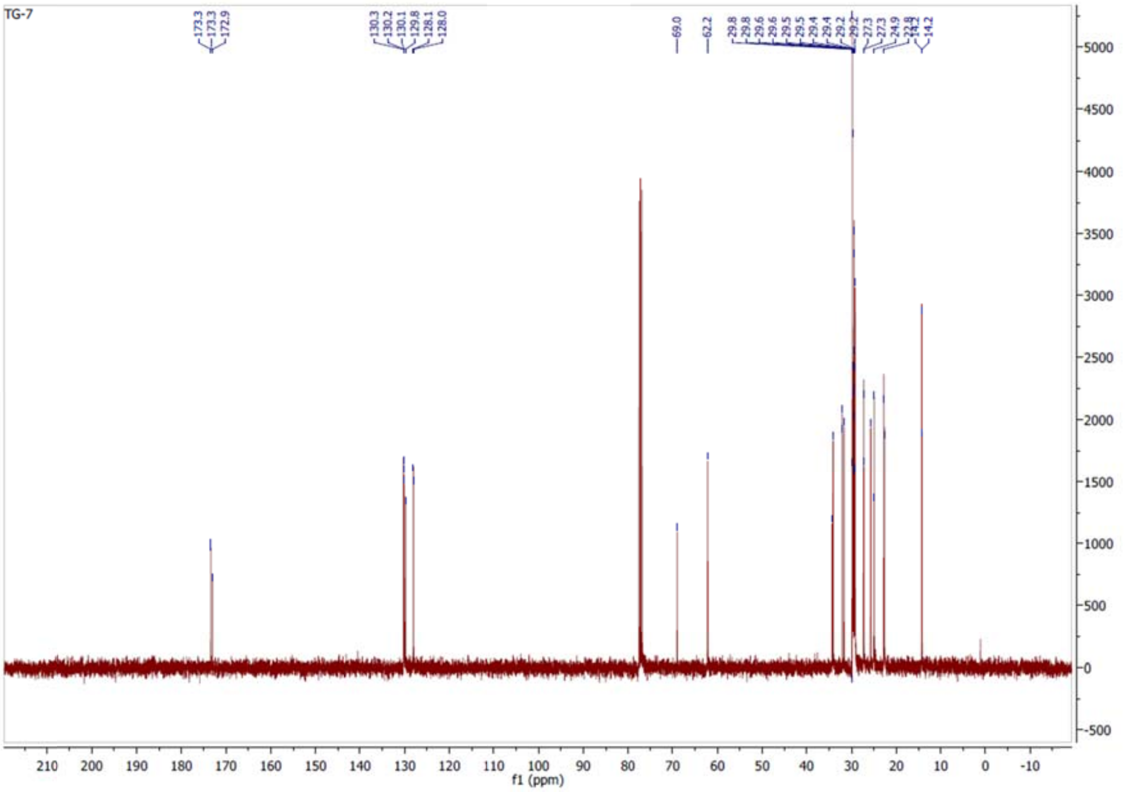

***2R*-TG (C20:2, 11z,14z; C16:0; C18:1, 9z), *(11Z,14Z)-(R)-3-(oleoyloxy)-2-(palmitoyloxy)propyl icosa-11,14-dienoate*, TG8.**

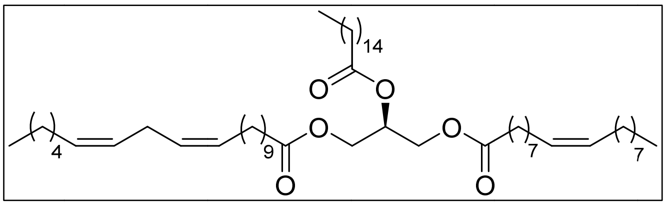

From 500 mg of (*S*)-(2,2-dimethyl-1,3-dioxolan-4-yl)methanol, following the **procedure A** with oleyl chloride, and **procedure B** to obtain **5b** with quantitative yield. **5b** is transformed following the **procedure C**, followed by **procedure A** with palmitoyl chloride to obtain, after purification by flash chromatography using Hexane/MTBE 1% as eluent, 1.16 g of **6b** (43% yield, 4 steps). 83 mg of **TG8** are obtained from 77 mg of **6b** following the **procedure D** and **procedure E** with (11Z,14Z)-icosa-11,14-dienoic acid after purification by flash chromatography using Hexane/MTBE 3% as eluent (87% yield, 2 steps). ^{1}H NMR (400 MHz, $CDCl_3$) δ 5.42 – 5.29 (m, 6H), 5.27 (tt, *J* = 5.9, 4.6 Hz, 1H), 4.29 (dd, *J* = 11.9, 4.3 Hz, 2H), 4.14 (dd, *J* = 11.9, 6.0 Hz, 2H), 2.77 (t, *J* = 6.5 Hz, 2H), 2.31 (td, *J* = 7.5, 2.4 Hz, 6H), 2.10 – 1.95 (m, 8H), 1.70 – 1.51 (m, 6H), 1.40 – 1.22 (m, 62H), 0.94 – 0.84 (m, 9H). ^{13}C NMR (101 MHz, $CDCl_3$) δ 173.3, 173.3, 173.0, 130.3, 130.2, 130.1, 129.8, 128.1, 128.0, 69.0, 62.2, 34.3, 34.2, 32.1, 32.0, 31.7, 29.9, 29.8, 29.8, 29.7, 29.7, 29.6, 29.5, 29.5, 29.4, 29.4, 29.4, 29.4, 29.3, 29.2, 29.2, 27.3, 27.3, 27.2, 25.7, 25.0, 24.9, 22.8, 22.7, 14.2, 14.2. Calcd for C57H104O6 (885,43 g/mol): C, 77.32; H, 11.84. Found: C, 77.22; H, 11.77%.

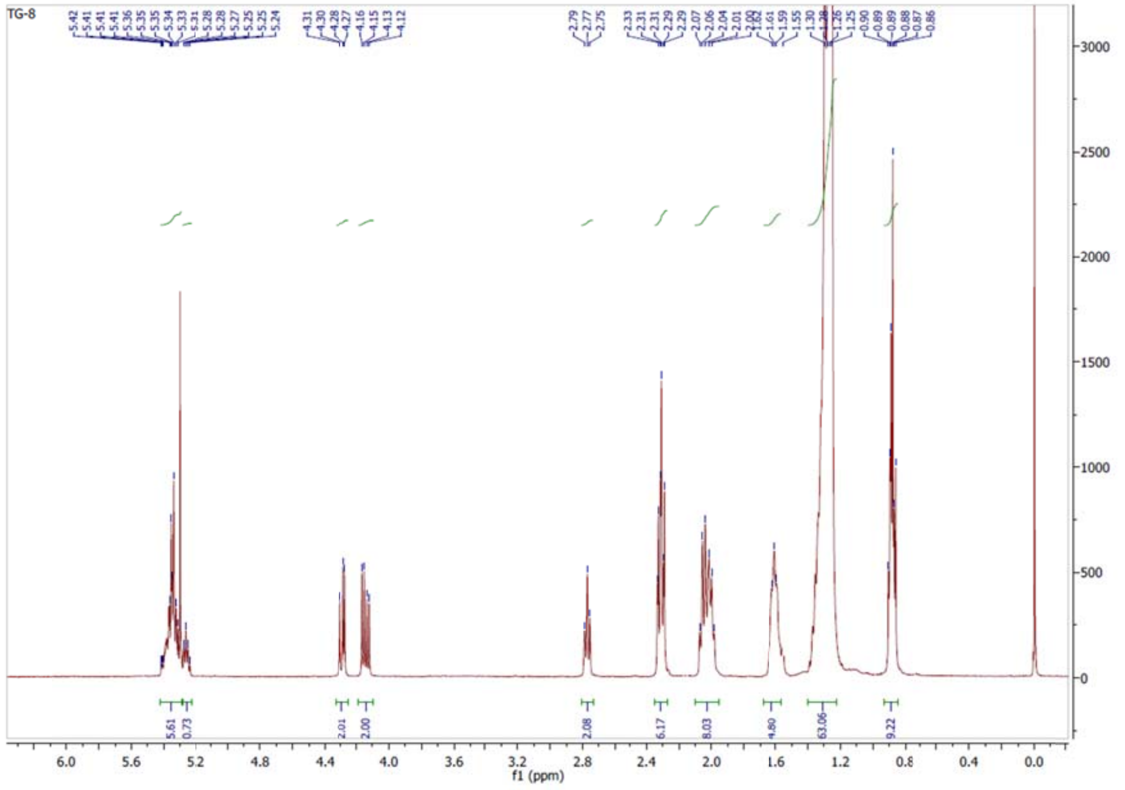
TG-8
f1 (ppm)

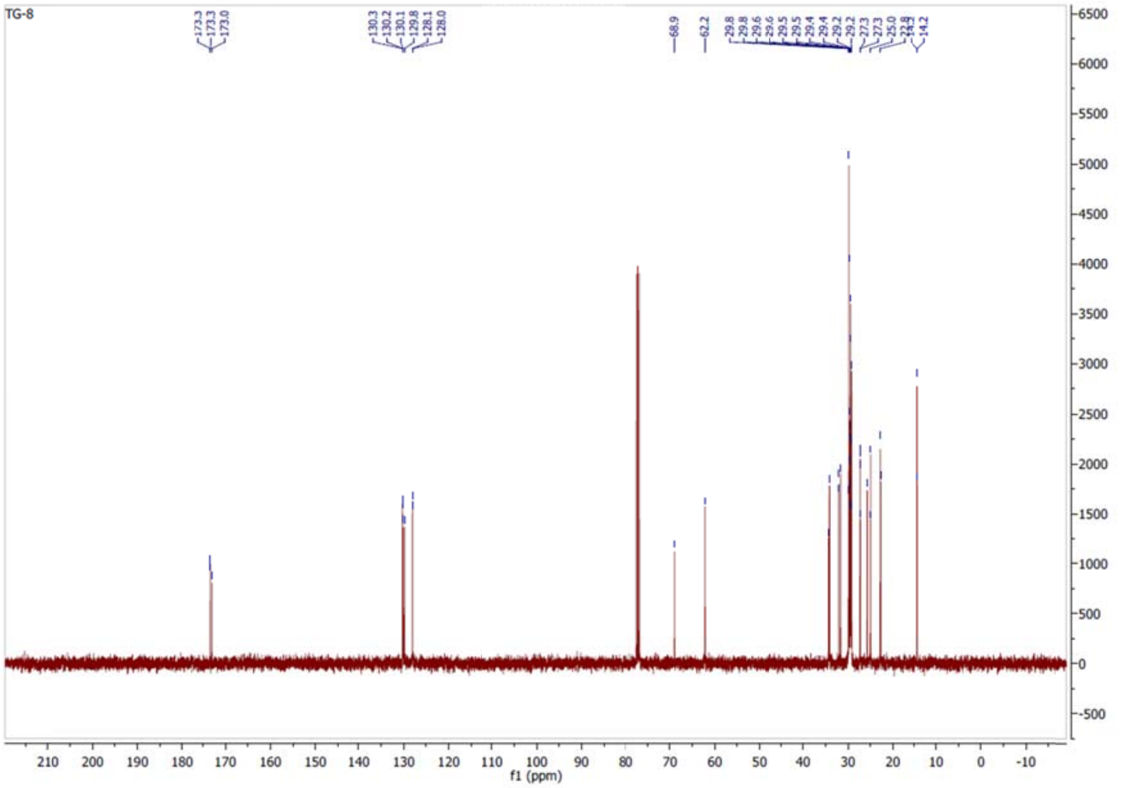
TG-8
f1 (ppm)

***2S*-TG (C18:2, 9z,12z; C16:0; C18:1, 9z), *(9Z,12Z)-(S)-3-(oleoyloxy)-2-(palmitoyloxy)propyl octadeca-9,12-dienoate*, TG9.**

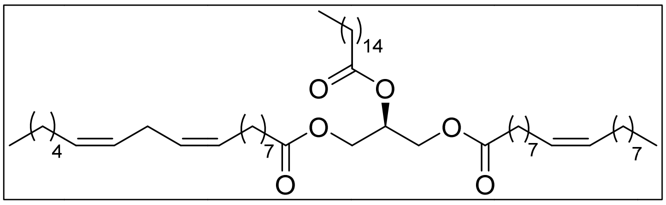

From 114 mg of **6a** following the **procedure D** and **procedure A** with linoleyl chloride after purification by flash chromatography using Hexane/MTBE 3% as eluent 104 mg of **TG9** are obtained (76% yield, 2 steps). ^{1}H NMR (400 MHz, $CDCl_3$) δ 5.42 – 5.29 (m, 6H), 5.29 – 5.23 (m, 1H), 4.29 (dd, J = 11.9, 4.3 Hz, 2H), 4.14 (dd, J = 11.9, 6.0 Hz, 2H), 2.77 (t, J = 6.6 Hz, 2H), 2.31 (td, J = 7.5, 2.1 Hz, 6H), 2.10 – 1.96 (m, 8H), 1.67 – 1.56 (m, 4H), 1.41 – 1.19 (m, 60H), 0.95 – 0.82 (m, 9H). ^{13}C NMR (101 MHz, $CDCl_3$) δ 173.3, 173.3, 172.9, 130.2, 130.1, 130.0, 129.7, 128.1, 127.9, 68.9, 62.1, 34.2, 34.1, 34.0, 32.0, 31.9, 31.5, 29.8, 29.7, 29.6, 29.6, 29.5, 29.5, 29.3, 29.3, 29.3, 29.3, 29.2, 29.1, 29.1, 27.2, 27.1, 25.6, 24.9, 24.8, 22.7, 22.6, 14.2, 14.1 Anal. Calcd for C55H100O6; C, 77.05; H, 11.76; (857,38 g/mol): Found: C, 76.85; H, 11.59%.

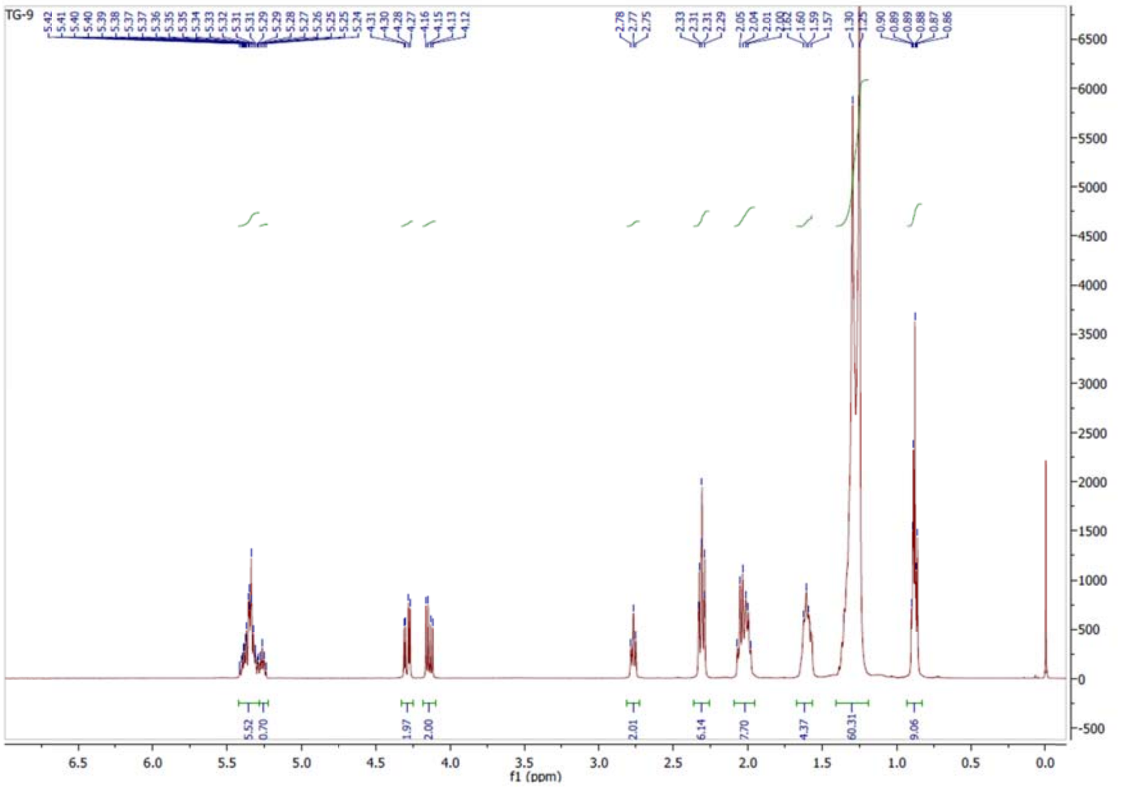

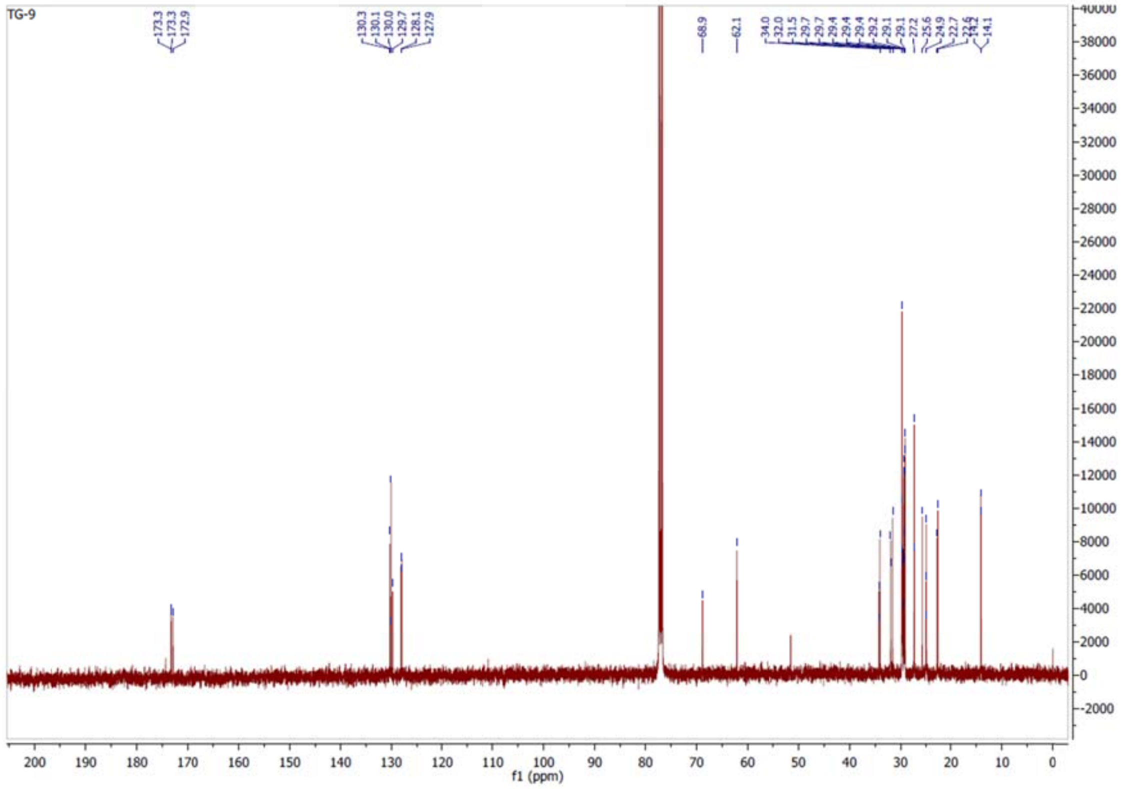

***2R*-TG (C18:2, 9z,12z; C16:0; C18:1, 9z), *(9Z,12Z)-(R)-3-(oleoyloxy)-2-(palmitoyloxy)propyl octadeca-9,12-dienoate*, TG10.**

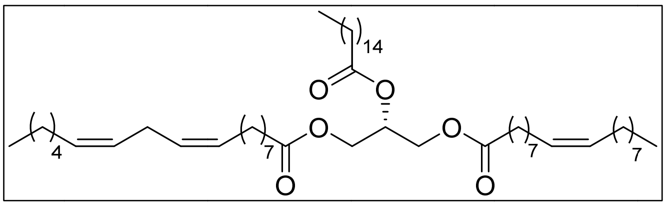

From 78 mg of **6b** following the **procedure D** and **procedure A** with linoleyl chloride after purification by flash chromatography using Hexane/MTBE 3% as eluent 94 mg of **TG10** are obtained (100% yield, 2 steps). ^{1}H NMR (400 MHz, $CDCl_3$) δ 5.42 – 5.29 (m, 6H), 5.29 – 5.23 (m, 1H), 4.29 (dd, *J* = 11.9, 4.3 Hz, 2H), 4.14 (dd, *J* = 11.9, 6.0 Hz, 2H), 2.77 (t, *J* = 6.4 Hz, 2H), 2.31 (td, *J* = 7.5, 2.1 Hz, 6H), 2.09 – 1.96 (m, 8H), 1.66 – 1.58 (m, 4H), 1.45 – 1.17 (m, 60H), 0.94 – 0.84 (m, 9H). ^{13}C NMR (101 MHz, $CDCl_3$) δ 173.3, 173.3, 172.9, 130.2, 130.1, 130.0, 129.7, 128.1, 127.9, 68.9, 62.1, 34.2, 34.1, 34.0, 32.0, 31.9, 31.5, 29.8, 29.7, 29.6, 29.6, 29.5, 29.5, 29.3, 29.3, 29.3, 29.3, 29.2, 29.1, 29.1, 27.2, 27.1, 25.6, 24.9, 24.8, 22.7, 22.6, 14.2, 14.1 Anal. Calcd for C55H100O6; C, 77.05; H, 11.76; (857,38 g/mol): Found: C, 76.92; H, 11.64%.

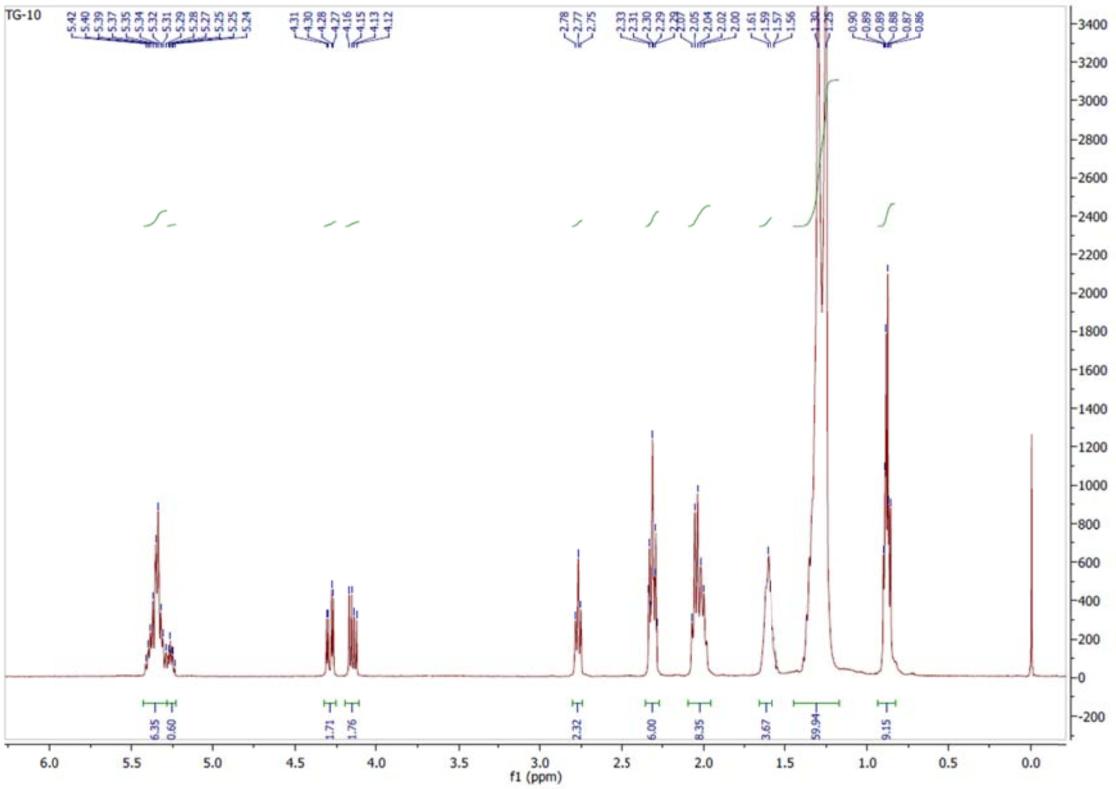
TG-10
5.42 5.40 5.39 5.37 5.35 5.34 5.32 5.31 5.29 5.28 5.27 5.25 5.25 5.24
4.31 4.30 4.28 4.27 4.16 4.15 4.13 4.12
2.78 2.77 2.75
2.33 2.31 2.30 2.29
2.07 2.05 2.04 2.02 2.00
1.61 1.59 1.57 1.56
1.30 1.25
0.90 0.89 0.89 0.88 0.87 0.86
6.35
0.60
1.71
1.76
2.32
6.00
8.35
3.67
59.94
9.15
f1 (ppm)

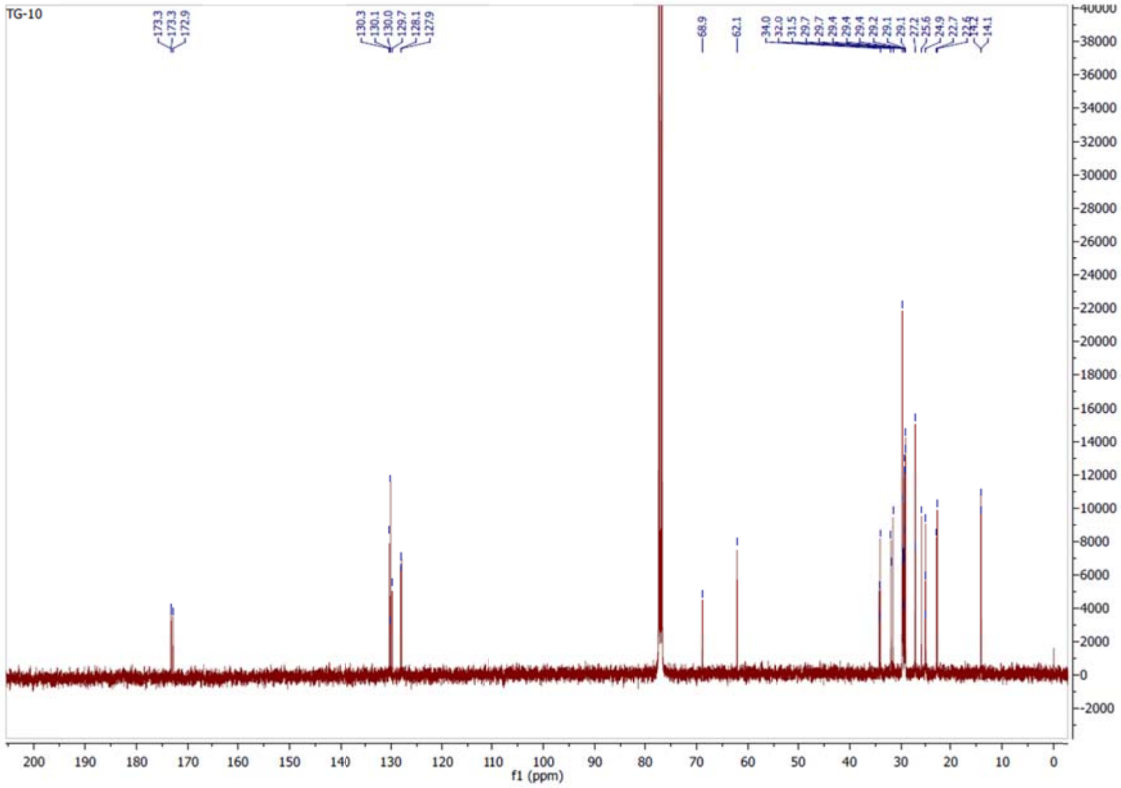
TG-10
173.3 173.3 172.9
130.3 130.1 130.0 129.7 128.1 127.9
68.9
62.1
34.0 32.0 31.5 29.7 29.7 29.4 29.4 29.2 29.1 29.1 29.1
27.2 25.6 24.9 22.7
14.1
f1 (ppm)

3. Growth modulation activity of synthetic triglycerides

Cell lines A549 (blue) and NL20 (red) were exposed to different concentration of the indicated triglycerides (TG) for 5 days, and the optical density (O.D.) was measured.

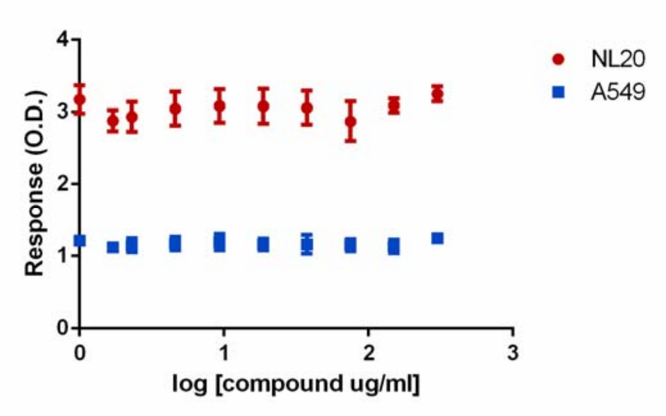

TG1

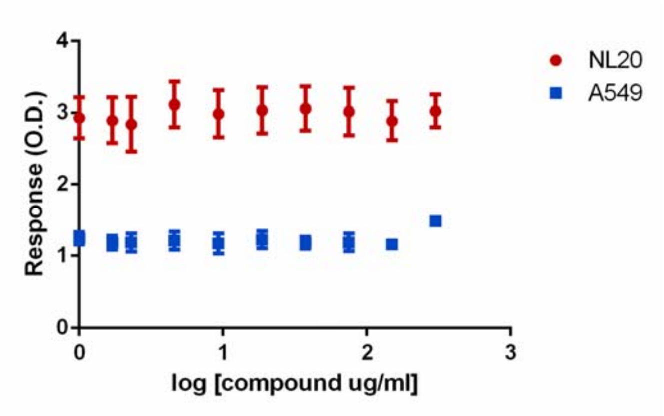

TG2

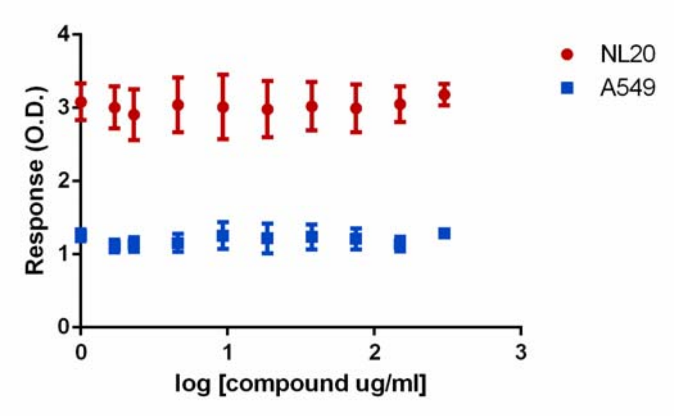

TG3

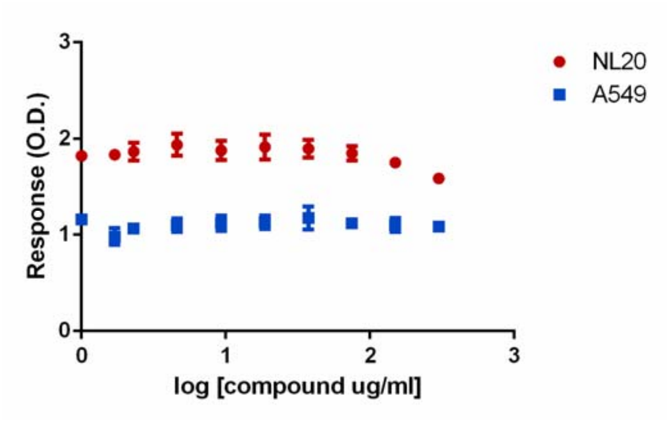

3
2
1
0
Response (O.D.)
0
1
2
3
log [compound ug/ml]
NL20
A549

TG4

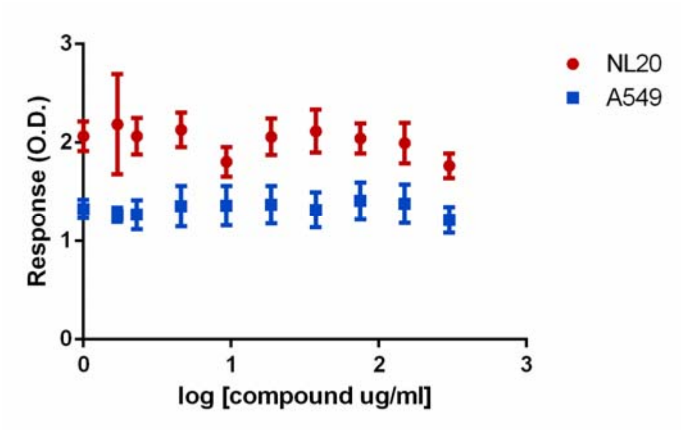

3
2
1
0
Response (O.D.)
0
1
2
3
log [compound ug/ml]
NL20
A549

TG5

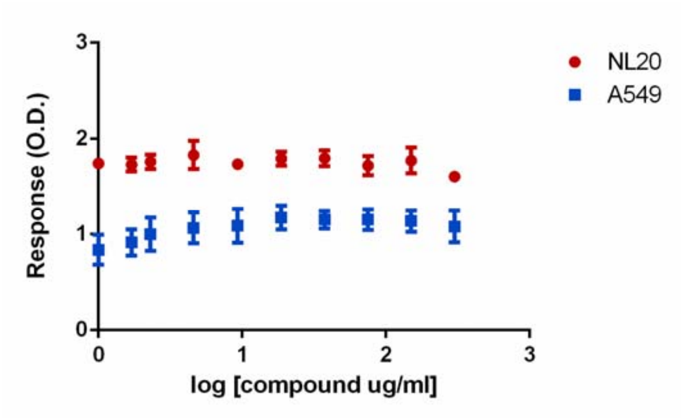

3
2
1
0
Response (O.D.)
0
1
2
3
log [compound ug/ml]
NL20
A549

TG6

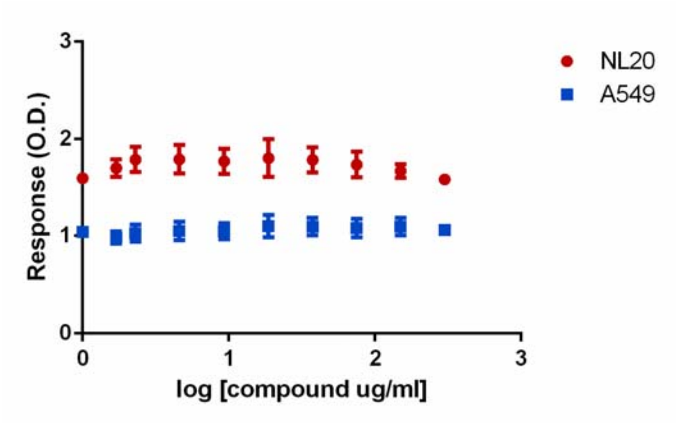

TG7

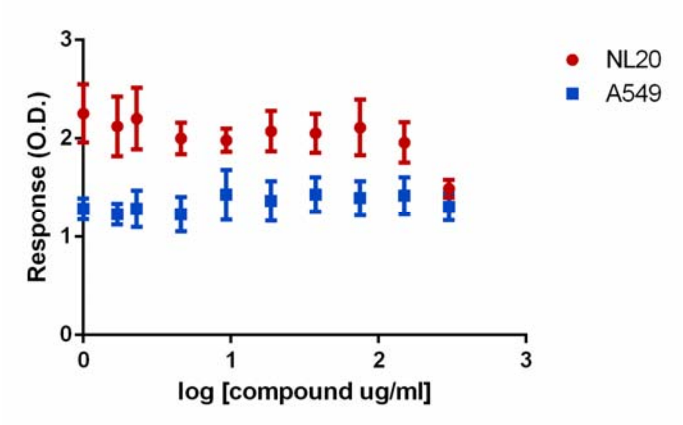

TG8

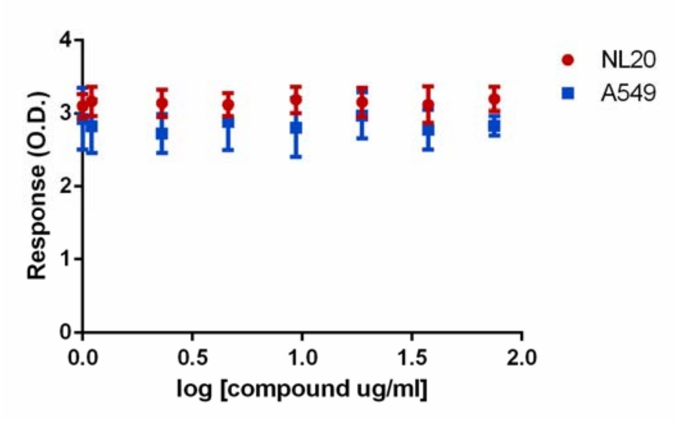

TG9

Results for **TG10** are shown in Figure 4 (main manuscript).

4. Comparison of ^{13}C-NMR spectra of aliphatic carbons between the synthetic triglyceride TG10 (red) and the natural extract (green)

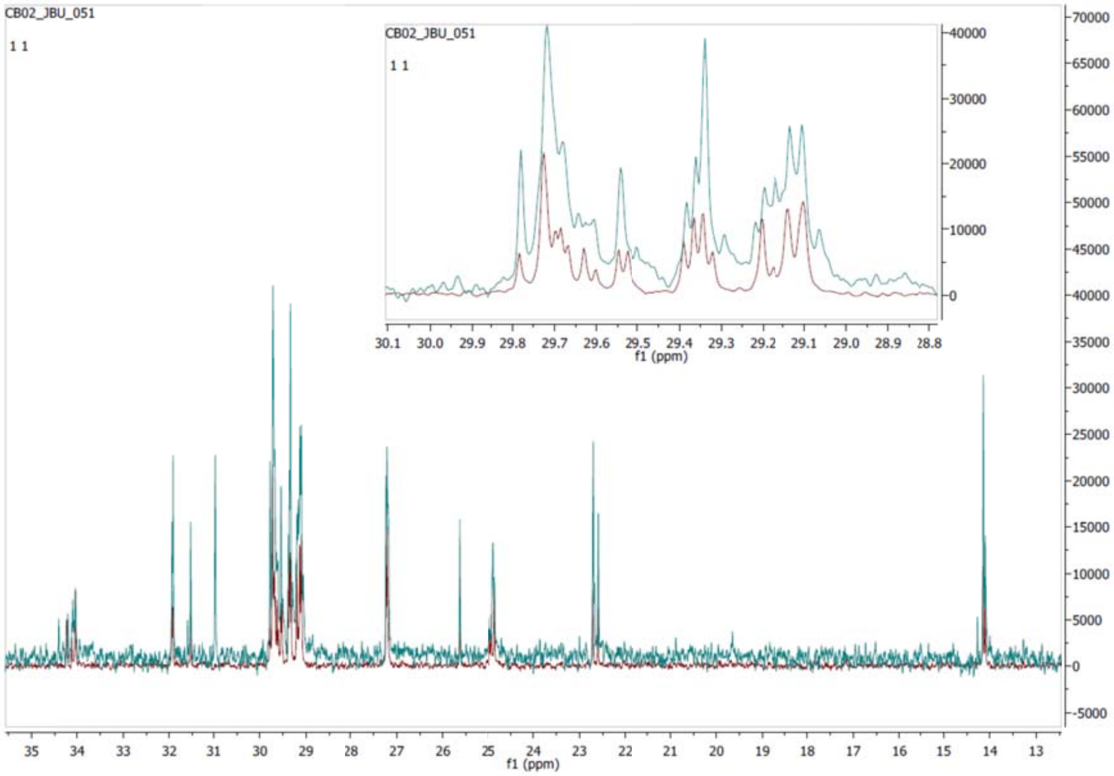

5. Dataset for xenograft study

Mice were injected with PBS as a control (AZ30-AZ45) or with Macrocybin (AZ26-AZ43) at the indicated days (d) after A549 tumor cell injection. Values represent tumor volume in mm3. Mouse AZ40 had to be sacrificed early because of a corneal ulcer.

Mouse codes	d14	d15	d18	d20	d22	d25
AZ30	95,57	75,83	91,54	83,77	139,81	279,34
AZ31	50,77	61,61	95,04	125,98	173,27	186,49
AZ32	55,87	84,48	125,79	109,28	146,66	181,39
AZ33	39,36	58,94	88,63	111,16	192,63	182,12
AZ34	24,02	11,07	32,97	167,27	198,16	235,59
AZ35	12,75	34,17	73,38	96,70	131,35	211,13
AZ36	55,62	91,79	92,61	132,30	116,04	284,34
AZ37	36,50	42,95	80,62	98,38	111,04	173,21
AZ44	10,05	23,72	36,62	50,46	80,18	127,93
AZ45	35,12	63,25	98,20	87,02	131,73	149,91
AZ26	53,68	99,04	123,99	191,01	221,17	267,96
AZ27	77,90	64,15	92,66	105,84	153,49	247,92
AZ28	31,13	27,73	27,68	24,57	44,92	44,96
AZ29	48,45	32,06	59,59	70,39	55,18	73,86
AZ38	42,69	49,37	104,25	105,66	130,25	195,44
AZ39	13,46	17,98	25,03	26,72	37,21	47,87
AZ41	42,39	44,37	60,34	94,85	150,74	170,39
AZ42	20,66	21,67	28,82	23,41	38,40	98,73
AZ43	23,07	38,30	60,49	81,87	81,90	159,54

d27	d29	d32	d34	d36	d39	d41
369,74	389,34	743,12	902,34	762,19	768,72	857,36
325,34	330,22	462,28	527,49	584,38	666,55	658,87
269,18	267,80	334,16	429,24	486,11	651,23	560,13
379,01	509,11	724,79	818,50	1108,49	1019,83	1140,44
259,53	286,32	644,73	830,32	839,56	858,94	1147,14
282,99	403,07	435,30	488,34	547,00	764,69	1072,99
177,84	483,79	908,66	913,59	1059,99	1032,26	1028,44
267,51	303,01	375,60	481,48	533,45	549,21	552,92
136,89	181,23	213,25	274,20	282,35	272,90	297,05
160,81	183,79	239,27	255,49	288,24	340,70	375,91
424,58	487,64	580,55	660,80	689,36	845,59	857,00
297,63	508,09	694,83	573,68	742,22	981,39	1087,49
39,94	72,26	99,72	134,73	97,36	185,48	182,20
117,74	120,27	131,38	171,26	157,68	209,00	183,03
211,87	228,24	310,53	307,82	292,13	376,58	428,24
45,51	69,49	132,41	112,55	90,93	150,94	153,75
207,31	196,98	254,95	282,18	412,03	624,50	540,00
80,90	104,52	190,06	196,01	230,97	301,75	333,86
179,28	209,38	233,72	306,08	371,39	500,67	494,62

d43	d46	d48	d50	d53	d55
1052,36	1099,10	1022,14	1303,67	1417,85	1655,35
606,17	546,98	694,16	1250,02	1027,84	1027,35
671,60	729,03	721,88	924,49		
1443,88	1381,97	1292,36	1765,38	2082,13	2417,64
1192,24	1548,99	1636,23	1786,41	1924,15	2123,91
889,24	1020,19	977,39	1438,55	1485,81	1612,99
1304,65	1281,01	1332,91	1464,66	1742,39	1614,96
651,90	605,06	757,74	688,57	710,65	669,92
326,41	410,77	431,96	500,24	617,04	637,09
408,57	422,91	466,54	488,15	563,10	619,44
857,50	892,86	937,38	1074,41	1065,86	
875,89	1041,49	974,87	1559,80	1326,90	1773,74
185,63	192,29	213,69	219,49	314,37	320,61
197,28	217,64	250,53	199,50	286,11	262,42
386,26	464,88	513,49	638,77	514,39	578,65
136,36	161,81	200,93	214,24	228,01	216,26
673,99	725,27	644,84	585,23	918,63	1035,26
369,27	473,34	501,03	637,15	839,82	825,99
572,41	418,51	671,53	855,05	814,50	844,37

6. Datasets for qRT-PCR study

The experiment was repeated 3 times (values are the quotient between Caveolin-1 and GAPDH expression).

<u>Experiment 1</u>

	A549 line			NL20 line		
Control	1,257057	1,219772	1,121098	1,115492	1,018236	0,9908198
TG10	1,445014	1,582141	1,609119	1,034146	0,9266025	1,031247

Sidak's multiple comparisons test	Mean Diff.	95% CI of diff.	Significant?	Summary	Adjusted P Value
TG10 - Control					
A549 line	0.3461	0.1079 to 0.5844	Yes	*	0.0143
NL20 line	-0.04418	-0.2824 to 0.1941	No	ns	0.8008

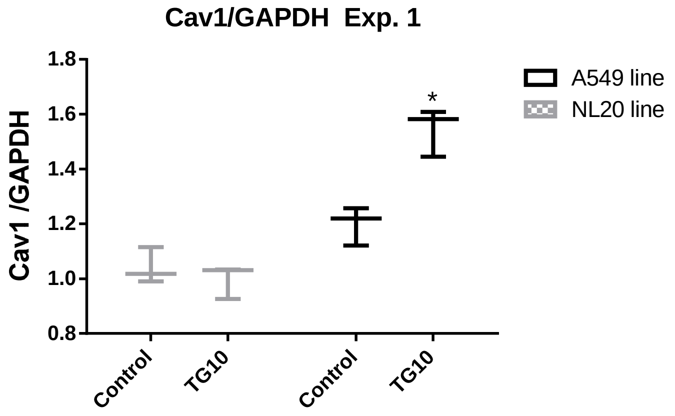

Table Analyzed	Cav1/GAPDH 201811				
Two-way RM ANOVA	Matching: Stacked				
Alpha	0.05				
Source of Variation	% of total variation	P value	P value summary	Significant?	
Interaction	19.11	0.0157	*	Yes	
tratamiento	11.44	0.0355	*	Yes	
celulas	62.52	0.0005	***	Yes	
Subjects (matching)	2.233	0.7557	ns	No	
ANOVA table	SS	DF	MS	F (DFn, DFd)	P value
Interaction	0.1143	1	0.1143	F (1, 4) = 16.26	P = 0.0157
tratamiento	0.06837	1	0.06837	F (1, 4) = 9.731	P = 0.0355
celulas	0.3737	1	0.3737	F (1, 4) = 112.0	P = 0.0005
Subjects (matching)	0.01335	4	0.003337	F (4, 4) = 0.4749	P = 0.7557
Residual	0.02810	4	0.007026		
Number of missing values	0				

Experiment 2

	A549 line						NL20 line					
Control	0,91497	0,97348	1,12069	1,23192	1,09348	1,10249	1,00372	1,14410	1,39692	1,25318	1,13059	1,13079
TG10	1,12692	1,24071	1,35905	1,31658	1,18018	1,17136	0,975355	1,13423	1,23951	1,17142	1,30580	1,14285

Sidak's multiple comparisons test	Mean Diff.	95% CI of diff.	Significant?	Summary	Adjusted P Value
TG10 - Control					
A549 line	0.1596	0.05158 to 0.2677	Yes	**	0.0061
NL20 line	-0.01502	-0.1231 to 0.09303	No	ns	0.9230

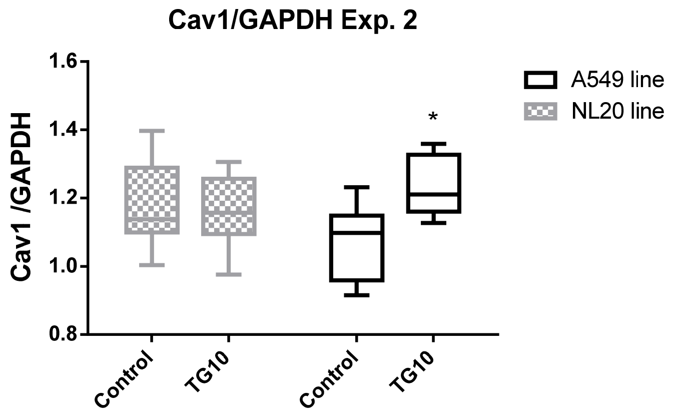

Table Analyzed	Cav1/GAPDH 202006 v2				
Two-way RM ANOVA	Matching: Stacked				
Alpha	0.05				
Source of Variation	% of total variation	P value	P value summary	Significant?	
Interaction	13.64	0.0133	*	Yes	
tratamiento	9.349	0.0322	*	Yes	
celulas	0.4802	0.7854	ns	No	
Subjects (matching)	61.40	0.0187	*	Yes	
ANOVA table	SS	DF	MS	F (DFn, DFd)	P value
Interaction	0.04575	1	0.04575	F (1, 10) = 9.011	P = 0.0133
tratamiento	0.03137	1	0.03137	F (1, 10) = 6.178	P = 0.0322
celulas	0.001611	1	0.001611	F (1, 10) = 0.07821	P = 0.7854
Subjects (matching)	0.2060	10	0.02060	F (10, 10) = 4.057	P = 0.0187
Residual	0.05077	10	0.005077		
Number of missing values	0				

Experiment 3

	A549 line						NL20 line					
Control	0,2190	0,2530	0,2810	0,2880	0,2960	0,2670	0,2410	0,2540	0,2760	0,2780	0,3040	0,2810
TG10	0,2740	0,3020	0,3150	0,2850	0,3130	0,2970	0,2350	0,2630	0,2940	0,2670	0,2850	0,2700

Sidak's multiple comparisons test	Mean Diff.	95% CI of diff.	Significant?	Summary	Adjusted P Value
TG10 - Control					
A549 line	0.03033	0.01104 to 0.04962	Yes	**	0.0041
NL20 line	-0.003333	-0.02262 to 0.01596	No	ns	0.8842

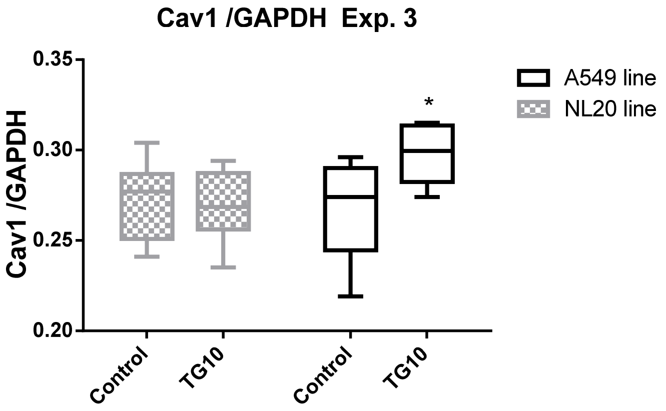

Table Analyzed	Cav1/GAPDH 20201104 v3				
Two-way RM ANOVA	Matching: Stacked				
Alpha	0.05				
Source of Variation	% of total variation	P value	P value summary	Significant?	
Interaction	12.68	0.0089	**	Yes	
tratamiento	8.157	0.0265	*	Yes	
celulas	6.267	0.3340	ns	No	
Subjects (matching)	60.82	0.0087	**	Yes	
ANOVA table	SS	DF	MS	F (DFn, DFd)	P value
Interaction	0.001700	1	0.001700	F (1, 10) = 10.51	P = 0.0089
tratamiento	0.001094	1	0.001094	F (1, 10) = 6.757	P = 0.0265
celulas	0.0008402	1	0.0008402	F (1, 10) = 1.030	P = 0.3340
Subjects (matching)	0.008154	10	0.0008154	F (10, 10) = 5.038	P = 0.0087
Residual	0.001618	10	0.0001618		
Number of missing values	0				
